# ATP and NADPH engineering of *Escherichia coli* to improve the production of 4-hydroxyphenylacetic acid using CRISPRi

**DOI:** 10.1186/s13068-021-01954-6

**Published:** 2021-04-20

**Authors:** Yu-Ping Shen, Yu-Ling Liao, Qian Lu, Xin He, Zhi-Bo Yan, Jian-Zhong Liu

**Affiliations:** 1 Institute of Synthetic Biology, Biomedical Center, Guangdong Province Key Laboratory of Improved Variety Reproduction in Aquatic Economic Animals, School of Life Sciences, Sun Yat-Sen University, Guangzhou, 510275 People’s Republic of China; 2 College of Chemistry and Bioengineering, Hunan University of Science and Engineering, Yongzhou, 425199 China

**Keywords:** 4-Hydroxyphenylacetic acid, ATP engineering, NADPH engineering, CRISPRi, *Escherichia coli*

## Abstract

**Background:**

4-Hydroxyphenylacetic acid (4HPAA) is an important raw material for the synthesis of drugs, pesticides and biochemicals. Microbial biotechnology would be an attractive approach for 4HPAA production, and cofactors play an important role in biosynthesis.

**Results:**

We developed a novel strategy called cofactor engineering based on clustered regularly interspaced short palindromic repeat interference (CRISPRi) screening (CECRiS) for improving NADPH and/or ATP availability, enhancing the production of 4HPAA. All NADPH-consuming and ATP-consuming enzyme-encoding genes of *E. coli* were repressed through CRISPRi. After CRISPRi screening, 6 NADPH-consuming and 19 ATP-consuming enzyme-encoding genes were identified. The deletion of the NADPH-consuming enzyme-encoding gene *yahK* and the ATP-consuming enzyme-encoding gene *fecE* increased the production of 4HPAA from 6.32 to 7.76 g/L. Automatically downregulating the expression of the *pabA* gene using the Esa-P_esaS_ quorum-sensing-repressing system further improved the production of 4HPAA. The final strain *E. coli* 4HPAA-∆yfp produced 28.57 g/L of 4HPAA with a yield of 27.64% (mol/mol) in 2-L bioreactor fed-batch fermentations. The titer and yield are the highest values to date.

**Conclusion:**

This CECRiS strategy will be useful in engineering microorganisms for the high-level production of bioproducts.

**Supplementary Information:**

The online version contains supplementary material available at 10.1186/s13068-021-01954-6.

## Background

4-Hydroxyphenylacetic acid (4HPAA), a valuable natural aromatic compound, has attracted much attention due to its numerous applications. 4HPAA is used in the synthesis of penicillin G, atenolol, benzoprofen, pesticides, etc. [1, 2]. 4HPAA is an effective ingredient of *Rhodiola rosea* [2] and the Chinese herbs *Aster tataricus* (fan hun cao). *A. tataricus* is widely used in the treatment of pneumonia, HBV and carcinomas in China [3–5]. Furthermore, 4HPAA has anxiolytic [6], antiplatelet [7] and hepatoprotective [8] properties. Moreover, 4HPAA is considered to be a potential hypopigmenting agent [9] and an inhibitor of hypertonicity and hypoxia [10].

4HPAA can be produced by chemical synthesis [11, 12]. However, this is energy intensive, environmentally unfriendly and expensive. To meet increasing market demand, heterologous biosynthesis in engineered microorganisms using synthetic biology and metabolic engineering provides an alternative way to produce 4HPAA. Koma et al. constructed *Escherichia coli* for the de novo production of 4HPAA from glucose by the overexpression of the *Azospirillum brasilense* NBRC102289 indole-3-pyruvate/phenylpyruvate decarboxylase gene *ipdC* and *E. coli* phenylacetaldehyde dehydrogenase gene *feaB* in a tyrosine-overproducing *E. coli* strain [1]. In our previous paper [13], applying a combinatorial strategy of the directed evolution of pathway enzymes and quorum-sensing-based dynamic regulation of the pathway further improved the production of 4HPAA, which reached 17.39 g/L in 2-L bioreactor fed-batch fermentation. However, the titer of 4HPAA in engineered microorganisms is much lower than that of other aromatic compounds [14]. Thus, further work is required to increase the production of 4HPAA.

The biosynthesis of metabolites often involves many oxidoreductases and kinases, which require cofactors, such as NADPH and ATP. For example, synthesizing one mol 4HPAA requires 2 mol ATP and 1 mol NADPH (Additional file 1: Fig. S1). Engineering cofactor availability is a common strategy of metabolic engineering for achieving high yields and productivity of metabolites. In *E. coli*, the pentose phosphate pathway (PPP), tricarboxylic acid cycle (TCA), and transhydrogenase systems are the three major sources of NADPH regeneration. The modulation of the three pathways has become a common method for increasing NADPH availability [15, 16]. Many ATP engineering strategies by metabolic engineering of pathways that generate or consume ATP have been successfully applied to the efficient production of chemicals [17]. However, these strategies may result in changes in the carbon central metabolism of *E. coli*.

In the *E. coli* genome, there are 80 NADPH-consuming and 400 ATP-consuming enzyme-encoding genes. Can the repression of these genes be used as a strategy for metabolic engineering? Recently, clustered regularly interspaced short palindromic repeat interference (CRISPRi) was developed for DNA sequence-specific gene regulation [18]. CRISPRi is a simple and useful tool for the downregulation of genes. It can effectively silence transcription elongation by targeting the promoter sequence or block transcription initiation by targeting the protein coding region (Additional file 1: Fig. S2). CRISPRi has been widely applied for metabolic engineering [18–23]. With the application of multiple gRNAs or a gRNA array, CRISPRi has been used for modulating multiple pathway genes simultaneously.

Thus, we developed a novel strategy called cofactor engineering based on CRISPRi screening (CECRiS). All NADPH-consuming and ATP-consuming enzyme-encoding genes of *E. coli* were repressed through CRISPRi, and the target genes that should be modified were identified to enhance the production of 4HPAA.

## Results and discussion

### Effects of NADPH-consuming enzyme-encoding genes

**Fig. 1:**
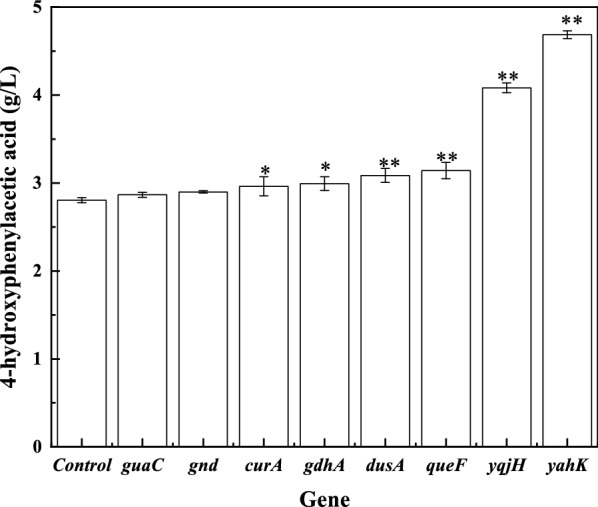
Effect of CRISPR interference of the selected NADPH-consuming enzyme- encoding genes on 4-hydroxyphenylacetic acid (4HPAA) production in *E. coli* 4HPAA-2. *E. coli* 4HPAA-2 harboring pCRISPathBrick* and pTargetB was set as the control. *P < 0.05 and **P < 0.01 relative the 4HPAA titer from the control. The data represent the means of three replicates, and error bars represent standard deviations

In the *E. coli* genome, 80 NADPH-consuming enzyme-encoding genes exist. To repress these genes, we constructed sgRNA-expressing plasmids of these genes and cotransferred with the dCas9* plasmid into the 4HPAA producer *E. coli* 4HPAA-2 for shake-flask analysis of 4HPAA production. The sgRNAs were designed to bind to the nontemplate DNA strands of the 5′ end of the gene (approximately 100 bp downstream of ATG) based on several previous studies [18, 21–23]. Strains expressing the sgRNA plasmids for *paaC, paaA, paaE, paaZ, paaB, panE, pdxI* and *ribD* could not grow (Additional file 1: Fig. S3), indicating that the repression of these genes significantly affected growth. Moreover, the repression levels of these genes using the sgRNAs targeting the 5′ end of the gene (approximately 100 bp downstream of ATG) may be too high to grow. To obtain the strain expressing the sgRNA plasmids for these genes, the N20 sequence should be redesigned to target the middle or 3′ end of the gene to reduce the repression level. As shown in Fig. 1 and Additional file 1: Fig. S3, repression of the *yahK, yqjH, queF, dusA, gdhA* and *curA* genes improved the production of 4HPAA in *E. coli* 4HPAA-2 by 67.1, 45.6, 11.9, 10.0, 6.8 and 5.3%, respectively. The *yahK* gene encodes NADPH-dependent aldehyde reductase, which catalyzes the reduction of a wide range of aldehydes into their corresponding alcohols. Koma et al. reported that YahK can convert 4-hydroxyphenylacetaldehyde into aromatic alcohol 2(4-hydroxyphenyl)ethanol [1]. This indicates that YahK can compete with 4HPAA biosynthesis for the consumption of 4-hydroxyphenylacetaldehyde. Thus, repression of the *yahK* gene increased the availability of 4-hydroxyphenylacetaldehyde for the production of 4HPAA. The *yqjH* gene encodes NADPH-dependent ferric siderophore reductase. YqjH has ferric reductase activity and is required for iron homeostasis in *E. coli* [24]. The *gdhA* gene encodes NADPH-dependent glutamine dehydrogenase, which catalyzes the reversible oxidative deamination of glutamate to alpha-ketoglutarate and ammonia. Alper et al. reported that knockout of the *gdhA* gene improved the production of lycopene by increasing the availability of NADPH [25].

### Effects of ATP-consuming enzyme-encoding genes

There are 400 genes encoding ATP-consuming enzymes in the *E. coli* genome*.* We constructed sgRNA-expressing plasmids of these genes and cotransferred them with the dCas9* plasmid into *E. coli* 4HPAA-2 to repress their expression for shake-flask analysis of 4HPAA production. As shown in Table 1 and Additional file 1: Fig. S4, CRISPRi-based repression of 19 genes resulted in an increase in the production of 4HPAA by 9–38%. These genes include *purC, araH, yeaG, sucC, dppD, artP, fecE, artM, argB, mgtA, aas, sapF, nanK, phnN, pfkA, ssuC, atpG, copA* and *hisP.* Of them, 9 genes encode transport protein genes, such as *araH, dppD, artP, fecE, artM, mgtA, sapF, ssuC* and *hisP.* In addition to increasing ATP availability, CRISPRi-based repressions may be beneficial to the transport of substrates, enhancing the production of 4HPAA. Recently, transporter engineering has been demonstrated to be a powerful strategy for improving the transmembrane transfer efficiency, protecting cells from toxic compounds, and enhancing microbial production [26]. The biosynthesis of arginine and aromatic amino acids requires glutamic acid. CRISPRi-based repression of the *argB* gene decreased the expression level of the biosynthetic pathway of arginine, increasing glutamic acid availability for 4HPAA production.

**Table 1:** Effect of CRISPR interference of the selected ATP-consuming enzyme-encoding genes on 4HPAA production in *E. coli* 4HPAA-2

Gene	Description	Ratio*
*purC*	Phosphoribosylaminoimidazole-succinocarboxamide synthase. It catalyzes the reaction: ATP + 5-amino-1-(5-phospho-D-ribosyl)imidazole-4-carboxylate + L-aspartate → ADP + 5′-phosphoribosyl-4-(N-succinocarboxamide)-5-aminoimidazole + phosphate + H^+^	1.38 ± 0.02
*araH*	Arabinose ABC transporter membrane subunit	1.36 ± 0.04
*yeaG*	Protein kinase	1.35 ± 0.03
*sucC*	Succinyl-CoA synthetase subunit β. It catalyzes the reaction: succinate + ATP + coenzyme A ↔ succinyl-CoA + ADP + phosphate	1.34 ± 0.01
*dppD*	Dipeptide ABC transporter ATP-binding subunit	1.33 ± 0.02
*artP*	L-Arginine ABC transporter ATP-binding subunit	1.33 ± 0.02
*fecE*	ferric citrate ABC transporter ATP-binding subunit	1.33 ± 0.03
*artM*	L-Arginine ABC transporter membrane subunit	1.30 ± 0.02
*argB*	Acetylglutamate kinase. It catalyzes the reaction: *N*-acetyl-L-glutamate + ATP → *N*-acetylglutamyl-phosphate + ADP	1.30 ± 0.02
*mgtA*	Mg^2+^ importing P-type ATPase	1.24 ± 0.02
*aas*	Acyltransferase. It has long-chain fatty acid-CoA/long-chain fatty acid [acyl-carrier-protein] ligase activity	1.22 ± 0.03
*sapF*	Putrescine ABC exporter ATP-binding protein	1.21 ± 0.02
*nanK*	N-Acetylmannosamine kinase. It catalyzes the reaction: *N*-acetyl-D-mannosamine + ATP → N-acetyl-D-mannosamine 6-phosphate + ADP + H^+^	1.20 ± 0.03
*phnN*	Ribose 1,5-bisphosphate phosphokinase, It catalyzes the reaction: α-D-ribose 1,5-bisphosphate + ATP ↔ 5-phospho-α-D-ribose 1-diphosphate + ADP	1.18 ± 0.02
*pfkA*	6-Phosphofructokinase 1. It catalyzes the reaction: β-D-fructofuranose 6-phosphate + ATP → ADP + β-D-fructose 1,6-bisphosphate + H^+^	1.18 ± 0.02
*ssuC*	Aliphatic sulfonates transport permease protein	1.17 ± 0.02
*atpG*	ATP synthase F1 complex subunit γ	1.15 ± 0.01
*copA*	Soluble Cu^+^ chaperone	1.11 ± 0.01
*hisP*	Histidine/lysine/arginine/ornithine transport ATP-binding protein	1.09 ± 0.03

The data represent the means of three replicates, and error bars represent standard deviations

To demonstrate the CRISPRi-based repression effect, we also analyzed the transcription levels of some genes after the CRISPRi-based repression. The sgRNAs were able to bind with their respective targets with the different efficiencies ranging from 63 to 80% repression due to the different genes (Additional file 1: Table 1). This result also demonstrates that the CRISPRi system is effective for gene repression.

Six NADPH-consuming and 19 ATP-consuming enzyme genes were identified using the CECRiS strategy. Moreover, these genes are not present in the 4HPAA biosynthetic pathway and are non-obvious target genes that could be manipulated for the enhanced production of the desired bioproduct. Of them, 9 genes (*araH, dppD, artP, fecE, artM, mgtA, sapF, ssuC* and *hisP*) encode transport protein genes. Recently, transporter engineering has been successfully used to improve the production of metabolites. Overexpression of transporters is a common strategy of transporter engineering for improving the production of the desired product [26]. However, there are some native transporters that facilitate the reuptake of final products, reducing the yield of microbial cells. Therefore, these native transporters must be inactivated to avoid product reuptake. Rodriguez et al. reported that the transcriptional levels of some transporter genes were downregulated [27]. The knockout of some of the transporters triggered a 20–50% improvement in the production of p-coumaric acid.

### Synergistic effects of NADPH-consuming and ATP-consuming enzyme-encoding genes

**Fig. 2:**
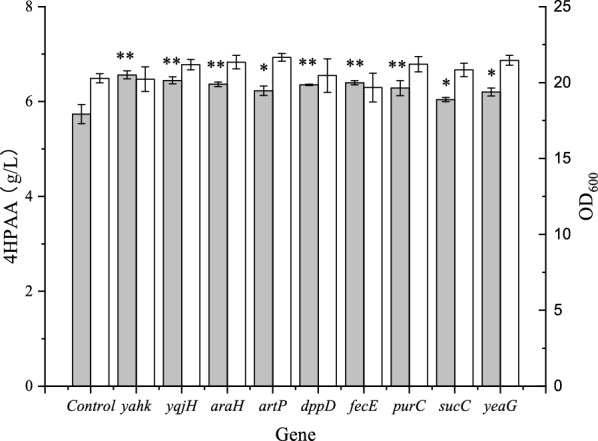
Effect of CRISPR interference of the selected genes on growth (white bar) and 4HPAA production (gray bar) in *E. coli* 4HPAA-GS-2–4. *E. coli* 4HPAA-2 harboring pCRISPathBrick* and pTargetB was set as the control. *P < 0.05 and **P < 0.01 relative the 4HPAA titer from the control. The data represent the means of three replicates, and error bars represent standard deviations

4HPAA has high toxicity to *E. coli.* Thus, we applied genome shuffling to obtain a shuffled strain *E. coli* 4HPAA-GS-2–4, with a higher tolerance to 4HPAA and titer (Additional file 1: Fig. S5). The above results demonstrate that CRISPRi-based repression of some genes encoding ATP- and NADPH-consuming enzymes improved the production of 4HPAA in *E. coli* 4HPAA-2. In order to determine whether the CRISPRi-based repression of these genes in the shuffled *E. coli* 4HPAA-GS-2–4 strain had the same results, we selected some genes whose 4HPAA titer was increased above 30% after CRISPRi in *E. coli* 4HPAA-2 and then investigated their effect in the shuffled strain *E. coli* 4HPAA-GS-2–4. As shown in Fig. 2, CRISPRi-based repression of *yahK, yqjH, araH, artP, dpp, fecE, purC, sucC* and *yeaG* in *E. coli* 4HPAA-GS-2–4 also increased the production of 4HPAA by 5%-14%. Additionally, CRISPRi-based repression did not affect growth, as shown in Fig. 2. This indicates that these genes can be deleted in the rest of the study.

**Fig. 3:**
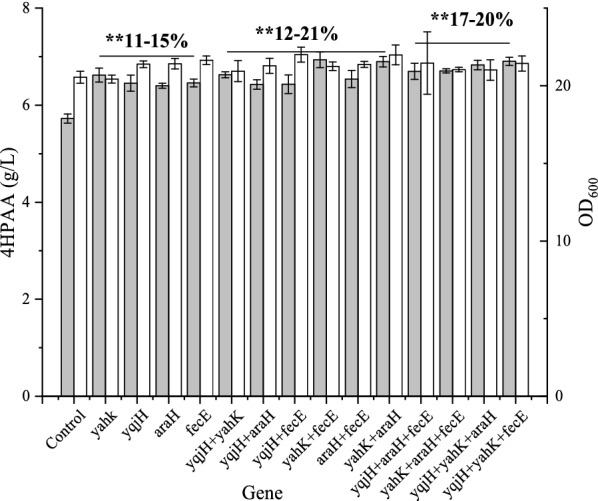
Effect of combined suppression of ATP- and NADPH-consuming enzyme-encoding genes on growth (white bar) and 4HPAA production (gray bar) in *E. coli* 4HPAA-GS-2–4. *E. coli* 4HPAA-GS-2-4 harboring pCRISPathBrick* and pTargetB was set as the control. **P < 0.01 relative the 4HPAA titer from the control. The data represent the means of three replicates, and error bars represent standard deviations

To investigate the synergistic effects of NADPH-consuming and ATP-consuming enzyme-encoding genes, two NADPH-consuming (*yahK* and *yqjH*) and ATP-consuming (*araH* and *fecE*) enzyme-encoding genes with the highest yield increase after CRISPRi-based repression were selected. We assembled the sgRNA-expressing vector harboring the N20 sequence of two genes. As shown in Fig. 3, the repression of the single, double and three genes increased the production of 4HPAA by 11–15, 12–21 and 17–20% (*P* < 0.01), respectively. Of them, the combined repression of the *yahK* and *fecE* had the strongest positive effect for the production of 4HPAA.

**Fig. 4:**
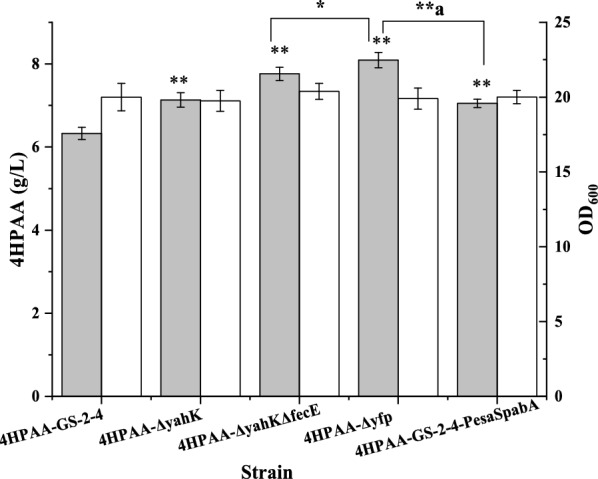
Effect of gene knockout on growth (white bar) and 4HPAA production (gray bar). *P < 0.05 relative the 4HPAA titer from 4HPAA--∆yahK ∆fecE. ** and **^a^*P* < 0.01 relative the 4HPAA titer from 4HPAA-GS-2–4 and 4HPAA-GS-2–4-P_esaS_-pabA, respectively. The data represent the means of three replicates, and error bars represent standard deviations

To avoid the metabolic burden caused by the dcas9- and sgRNA-expressing vectors, *yahK* and *fecE* in *E. coli* 4HPAA-GS-2–4 were deleted, and the production of the deletion strains was assayed. As shown in Fig. 4, the deletion of the NADPH-consuming enzyme-encoding gene *yahK* increased the production of 4HPAA to 7.13 ± 0.17 from 6.32 ± 0.15 g/L. Moreover, the 4HPAA titer of the *yahK* knockout was slightly higher than that obtained by the *yahK* knockdown. It may be because the CRISPRi cannot complete repression the expression of the *yahK* (only 63% repression, Additional file 1: Table 1). The deletion of the ATP-consuming enzyme-encoding gene *fecE* in the *yahK* knockout further improved the production of 4HPAA to 7.76 ± 0.16 g/L.

### Dynamic regulation of an essential gene using quorum-sensing (QS)-repressing system

**Fig. 5:**
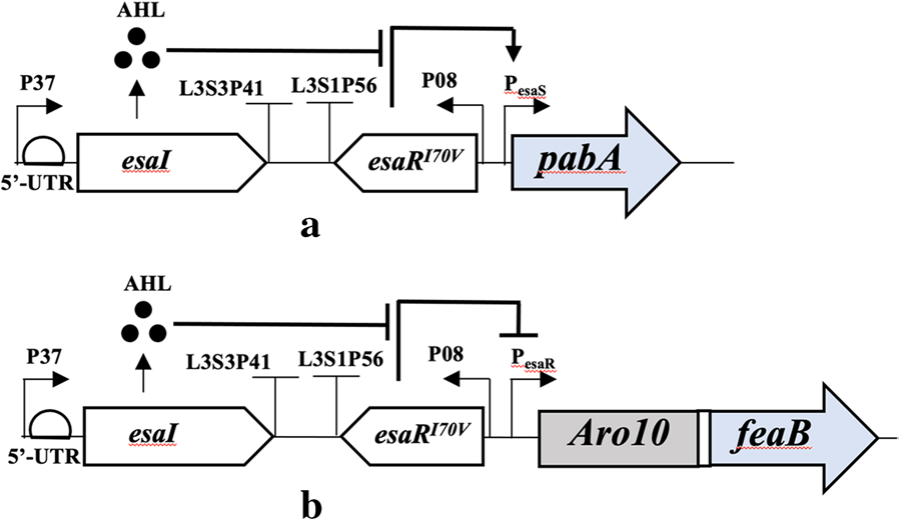
Quorum-sensing (QS) regulation system in *E. coli* 4HPAA-*△yfp*. **a** The Esa-P_esaS_ QS-repressing system for automatically downregulating the expression of the *pabA* gene based on cell density. At low cell density, the *pabA* gene controlled by P_esaS_ expresses. At high cell densities, the *pabA* gene controlled by P_esaS_ does not express. **b** The Esa-P_esaR_ QS activation system for automatically upregulating the expression of the heterologous 4HPAA biosynthetic pathway. At low cell density, the 4HPAA biosynthetic pathway gene cluster controlled by P_esaR_ does not express. At high cell densities, the 4HPAA biosynthetic pathway gene cluster controlled by P_esaR_ expresses

Yang et al. reported that the *pabA* gene encoding aminodeoxychorismate synthase subunit 2 should be knocked down for L-tyrosine derivatives phenylpropanoid (resveratrol and naringenin) production using synthetic small regulatory RNAs [28]. PaBA catalyzes the synthesis of aminobenzoate from chorismate involved in the tetrahydrofolate (vitamin B9) biosynthetic pathway. It also competes for chorismate with 4HPAA biosynthesis. Moreover, synthesizing tetrahydrofolate from chorismate also requires consuming both ATP and NADPH. Thus, we investigated whether knocking down or out this gene also improves the production of 4HPAA. Deleting this gene results in a tetrahydrofolate auxotrophic phenotype. To overcome this problem, we applied an Esa QS-repressing system to downregulate the expression of the *pabA* gene. In our previous paper [13], we reported an Esa QS-repressing system from *Pantoea stewartii* that consists of *esaI*, *esaR*^I70V^ and the P_esaS_ promoter. The native promoter of the *pabA* gene in *E. coli* 4HPAA-∆yahK∆fecE was replaced with the Esa-P_esaS_ QS-repressing system to obtain *E. coli* 4HPAA-∆yfp. The concentration of the QS signaling molecule (3-oxohexanoyl-homoserine lactone, AHL) produced by the AHL synthase EsaI is low at low cell density and increases with the cell density. At low cell density, P_esaS_ is activated by EsaR^I70V^, driving the *pabA* gene expression. As cell density increases, the AHL accumulates, resulting in disruption of the transcriptional regulator EsaR^I70V^ binding and repression of the transcription of the *pabA* gene (Fig. 5a). In other words, the transcription level of the *pabA* decreased with cell growth. As shown in Fig. 4, knocking down the *pabA* gene with the Esa-P_esaS_ QS-repressing system in *E. coli* 4HPAA--∆yahK ∆fecE further improved the production of 4HPAA to 8.09 ± 0.18 from 7.76 ± 0.16 g/L (*P* < 0.05). To avoid the aromatic amino acid auxotrophic phenotype caused by the deletion of shikimate kinase encoded by *aroK* and *aroL*, the Esa-P_esaS_ QS-repressing system from *P. stewartii* was also used to dynamically downregulate AroK to increase the titer of shikimate in *E. coli* from a previously unmeasurable amount to 105 mg/L [29]. In the *aroK* and *aroL* knockout strain, the *aroK* transcription was dynamically downregulated using the Esa-P_esaS_ QS-repressing system with cell growth. Therefore, this resulting strain can grow in minimal medium without aromatic amino acid supplementation and accumulate shikimate.

To avoid the auxotrophic phenotype caused by the knockout of the *pabA* gene, the Esa-P_esaS_ QS-repressing system was used to automatically downregulate the expression of the *pabA* gene based on cell density in this study. This QS-repressing system was also used to dynamically downregulate the competitive glycolysis pathway, resulting in increases in the production of myo-inositol and glucaric acid of up to 5.5- and 4-fold, respectively [29]. Our final *E. coli* 4HPAA-∆yfp strain has a bifunctional QS system (Fig. 5). The Esa-P_esaR_ QS activation system was used to automatically upregulate the expression of the heterologous 4HPAA biosynthetic pathway (Fig. 5b), and the Esa-P_esaS_ QS-repressing system was used to automatically downregulate the expression of the *pabA* gene based on cell density (Fig. 5a). This bifunctional QS system was also applied to the production of 5-aminolevulinic acid and poly-b-hydroxybutyrate, leading to 6- and 12-fold titers, respectively [30].

**Fig. 6:**
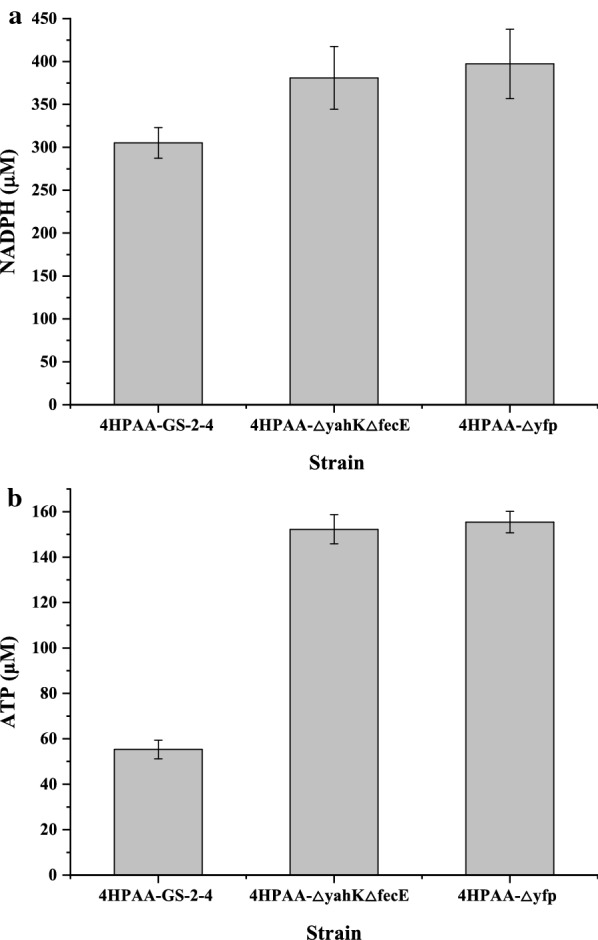
Intracellular ATP (**a**) and NADPH (**b**) concentrations of the selected strain. The data represent the means of three replicates, and error bars represent standard deviations

To investigate the relationship between ATP/or NADPH availability and the production of 4HPAA, we also assayed the intracellular ATP and NADPH concentrations. As shown in Fig. 6, the double knockouts of *yahK* and *fecE* increased the intracellular NADPH and ATP concentrations to 380.93 ± 36.52 and 152.22 ± 6.39 µM from 305.28 ± 17.82 and 55.30 ± 4.13 μM, respectively. The knockdown of the *pabA* gene with the Esa-P_esaS_ QS-repressing system further increased the intracellular NADPH and ATP concentrations. This is because tetrahydrofolate biosynthesis from chorismate requires NADPH, ATP and glutamate. Moreover, the dynamic downregulation of the *pabA* gene also increased glutamate availability for the production of 4HPAA.

**Fig. 7:**
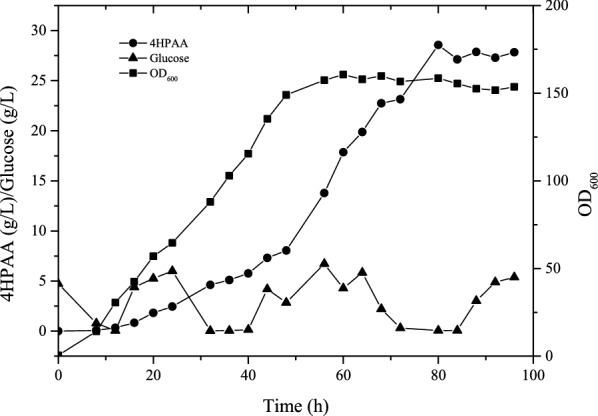
Fed-batch fermentation of *E. coli* 4HPAA-*△yfp*. Growth (■); 4HPAA concentration (●); glucose concentration (▲). Experiments were conducted in duplicate, and measurements are presented as the means with standard deviations

To evaluate the 4HPAA production of *E. coli* 4HPAA-∆yfp, fed-batch was performed in a 2-L bioreactor (Fig. 7). This engineered strain produced the highest level of 4HPAA of 28.57 g/L with a yield of 27.64% (mol/mol) at 72 h. This titer and yield were higher than those reported in our previous paper [13]. As shown in Fig. 7, the OD_600_ value reached 160.65, which is an approximately threefold increase compared to values reported in our previous paper [13]. This is because this strain is derived from the 4HPAA-tolerant strain *E. coli* 4HPAA-2.

## Conclusion

We developed a CECRiS strategy for the metabolic engineering of microorganisms. Some target genes were first identified and then deleted or knocked down using the Esa-P_esaS_ QS-repressing system. This strategy was used to improve NADPH and/or ATP availability, enhancing the production of 4HPAA. After CRISPRi screening, 6 NADPH-consuming and 19 ATP-consuming enzyme genes were identified. The deletion of the NADPH-consuming enzyme-encoding gene *yahK* and the ATP-consuming enzyme-encoding gene *fecE* increased the production of 4HPAA by 22.8%. Automatically downregulating the expression of the *pabA* gene using the Esa-P_esaS_ QS-repressing system further improved the production of 4HPAA. The final strain *E. coli* 4HPAA-∆yfp produced 28.57 g/L 4HPAA with a yield of 27.64% (mol/mol) after 72 h of fermentation under fed-batch conditions. The titer and yield are the highest values to date. This CECRiS strategy will be useful in engineering microorganisms for the high-level production of bioproducts.

## Methods

### Strains, plasmids and primers

The bacterial strains and plasmids used in this study are listed in Table 2. The primers used in this study are presented in Additional file 1: Table 2.

**Table 2:** Strains and plasmids used in this study

Name	Description		Source/purpose
Strain			
*E. coli* DH5α	*supE44* Δ(*lacZYA-argF*) *U169* (Φ*80lacZ* Δ*M15*) *hsdR17 recA endA1 gyrA96 thi-1 relA1*		Invitrogen
*E. coli* 4HPAA-2	4-Hydroxyphenylacetic acid (4HPAA) producer, *E. coli* DOPA-30 N, *ΔSS9::P*_*37*_-*esaI*-*P*_*08*_*-esaR*^*I70V*^-P_esa_S -P_esaR_-aro10*-TIGR-feaB*,ΔhpaBC:: P_*37*_-*esaI*-*P*_*08*_*-esaR*^*I70V*^-P_esa_S -P_esaR_-aro10*-TIGR-feaB*		Lab stock
*E. coli* 4HPAA-GS-2–4	4HPAA overproducer tolerant to 35.0 g/L 4HPAA with higher 4HPAA titer than *E. coli* 4HPAA-2 obtained after ARTP mutagenesis and genome shuffling		Lab stock
*E. coli* 4HPAA--∆yahK	4HPAA overproducer, *E. coli* 4HPAA-GS-2–4, -∆yahK		This study
*E. coli* 4HPAA--∆yahK ∆fecE	4HPAA overproducer, *E. coli* 4HPAA-GS-2–4,-∆yahK* ,*∆fecE		This study
*E. coli* 4HPAA-GS-2–4-P_esaS_-pabA	4HPAA overproducer, *E. coli* 4HPAA-GS-2–4, P_pabA_:: *P*_*37*_-*esaI*-*P*_*08*_*-esaR*^*I70V*^-P_esa_S		This study
*E. coli* 4HPAA-∆yfp	4HPAA overproducer, *E. coli* 4HPAA-GS-2–4, -∆yahK* , *∆fecE* ,* P_pabA_:: *P*_*37*_-*esaI*-*P*_*08*_*-esaR*^*I70V*^-P_esa_S		This study
Plasmid			
pCRISPathBrick*	*E. coli dcas9* (K848A/K1003A/ R1060A) expression vector		[31, 32]
pCas*	*E. coli cas9* (K848A/K1003A/ R1060A) expression vector		[31]
pTargetB	*E. coli* sgRNA expression vector, BglBrick vector		[33]

### Construction of the CRISPRi system

A CRISPRi system was used to repress gene expression as previously described [23].

The N20 sequence was designed to target the 5′ end of the gene (approximately 100 bp downstream of ATG) on the nontemplate DNA strand as previously described [18, 21–23]. sgRNA-X was amplified from pTargetB (Additional file 1: Fig. S6) using primers *Spe*I-TargetX/*EcoR*I-*Bgl*II-TargetR and then inserted into the *Spe*I/*EcoR*I sites of pTargetB to obtain the sgRNA plasmid pTargetB-X. pCRISPathBrick* (Additional file 1: Fig. S6) and pTargetB-X were cotransferred into *E. coli* cells for the repression of genes.

### Knockout or replacement of gene

Gene knockout or replacement was performed according to the CRISPR–Cas method as previously described [23, 31, 34]. The sgRNA plasmid pTargetB-X for knockout or replacement was obtained as described above for the CRISPRi system. The target fragment for knockout or replacement was amplified by overlap PCR and then inserted into *EcoR*I/*Sal*I-digested pTargetB-X to obtain pTargetB-X. pCas* and pTargetB-X were cotransferred into *E. coli* cells to knock out or replace the corresponding gene.

### Production of 4HPAA

A single colony was picked from the plates and grown in a Falcon tube containing 5 mL of LB medium at 30 °C and 200 rpm overnight. The resulting seed culture was then inoculated into 250-mL flasks containing 50 mL of fermentation medium (10 g/L tryptone, 5 g/L yeast extract, 10 g/L NaCl, 40 g/L glucose, 0.6 g/L KH_2_PO_4_, 2.56 g/L Na_2_HPO_4_·7H_2_O and 10 mL/L trace element solution) with a starting OD_600_ of 0.1. The trace element solution contained (per liter) 10 g of FeSO_4_·7H_2_O, 2.2 g of ZnSO_4_·7H_2_O, 0.58 g of MnSO_4_·4H_2_O, 1 g of CuSO_4_·5H_2_O, 0.1 g of (NH_4_)_6_Mo_7_O_24_·4H_2_O and 0.2 g of Na_2_B_4_O_7_·10H_2_O. The pH of the medium was adjusted to 7.0. The main cultures were incubated at 30 °C and 200 rpm for 72 h.

Fed-batch fermentation was performed in a 2-L fermenter (MiniBox 2 L*2 Parallel Bioreactor System, T&J Bioengineering (Shanghai) Co. LTD, Shanghai, China) containing 1.2 L of fermentation medium with an initial OD_600_ of approximately 0.1. The temperature was controlled at 30 °C, and the pH value was maintained at 7.0 by the automatic addition of NH_4_OH. The airflow rate was 1.2 L/min. Dissolved oxygen was kept above 25% by adjusting the agitation rate from 400 to 1200 rpm. A feed solution (pH 7.0) containing (per liter) 500 g glucose and 30 g MgSO_4_·7H_2_O was fed continuously to the fermenter using a pH-stat feeding strategy. Once the glucose was depleted, the pH rose rapidly. When the pH was greater than 7.1, the feed was automatically added to the fermenter. Samples were periodically withdrawn, and these parameters (OD_600_, residual glucose concentrations and 4HPAA concentrations) were determined.

### Assay

Cell density was monitored by measuring the optical density at 600 nm. 4HPAA in the supernatants was analyzed using an HPLC system (LC-20A, Shimadzu, Japan) with a photodiode array detector (SPD-M20A) at 222 nm using an Inertsil ODS-SP column (5 μm, 4.6 × 150 mm, GL Sciences Inc., Tokyo, Japan), which was kept at 30 °C. The mobile phase was 0.2% TFA in methanol at a flow rate of 0.5 mL/min. The methanol concentration was increased from 14 to 45% for 20 min, decreased to 14% and then maintained for 10 min. The concentration of 4HPAA was quantified by the standard curve method. Glucose concentrations were determined using a glucose assay kit (Shanghai Rongsheng Biotech Corporation, Shanghai, China) with glucose oxidase.

### Assay of intracellular NADPH concentration

The intracellular NADPH concentration was measured by HPLC as previously described [33, 35]. The cells cultured for 44 h were immediately chilled in an ice bath, centrifuged at 5200 × g for 10 min and then resuspended in 1 mL of deionized water to a final OD_600_ of 30. Then, 500 µL of 0.3 M NaOH was added, incubated at 60 °C for 10 min, and neutralized by 500 µL of 0.3 M HCl and 100 µL of Tricine–NaOH (pH 8.0). The neutralized samples were centrifuged at 12,000 × g and 4 °C for 20 min, and the resulting supernatants were filtered through a 0.22-μm membrane. The concentrations of NADPH were determined using an HPLC system (LC-20A HPLC, Shimadzu, Japan) equipped with an Inertsil ODS-SP column (5 μm, 4.6 × 150 mm, GL Sciences Inc., Tokyo, Japan), which was kept at 30 °C and detected at 254 nm. The mobile phase composed of A (0.2 M phosphate buffer containing 10 mM tetrabutylammonium bromide, pH 7.0) and B (methanol) at a ratio of 80:20 with a flow rate of 0.8 mL/min. The concentration of NADPH was quantified by the standard curve method.

### Assay of intracellular ATP concentration

The intracellular ATP concentration was determined by HPLC [36]. The cells cultured for 44 h were immediately cooled in an ice bath, centrifuged at 5200 × g for 10 min, resuspended in 6% perchloric acid to a final OD_600_ of 30, ultrasonically broken in an ice bath and then neutralized with 0.6 mL of saturated K_2_CO_3_. The solution was then centrifuged at 12,000 × g and 4 °C for 20 min, and the resulting supernatants were filtered through a 0.22-μm membrane. The concentrations of ATP were determined using an HPLC system (LC-20A HPLC, Shimadzu, Japan) equipped with an Inertsil ODS-SP column (5 μm, 4.6 × 150 mm, GL Sciences Inc., Tokyo, Japan), which was kept at 30 °C and detected at 254 nm. The mobile phase was phosphate buffer containing 0.06 M K_2_HPO_4_ and 0.04 M KH_2_PO_4_ (pH 7.0) with a flow rate of 1.0 mL/min. The concentration of ATP was quantified by the standard curve method.

### Statistical analysis

All experiments were carried out three times, and the data were taken as the means ± standard deviation. Significant differences were determined by Tukey’s test using the OriginPro (version 7.5) package. Statistical significance was defined as *p* < 0.05.

## Supplementary Information

**Additional file 1**: **Figure S1** Biosynthetic pathway of 4-hydroxyphenylacetic acid in the engineered Escherichia coli used in this study. Black: E. coli genes; red: Saccharomyces cerevisiae gene. PEP: phosphoenolpyruvate; E4P: D-erythrose 4-phosphate; DAHP: 3-deoxy-arabino-heptulonate 7-phosphate; DHQ: 3-dehydroquinate; DHS: 3-dehydroshikimate; EPSP: enolpyruvoyl-shikimate 3-phosphate. aroF/aroH/aroG: 3-deoxy-7-phosphoheptulonate synthase gene; aroB: 3-dehydroquinate synthase gene; aroD: 3-dehydroquinate dehydratase gene; aroE: shikimate dehydrogenase gene; aroK/aroL: shikimate kinase gene; aroA: 3-phosphoshikimate 1-carboxyvinyltransferase gene; aroC: chorismate synthase gene; tyrA: chorismate mutase/prephenate dehydrogenase gene; pheA: chorismate mutase/prephenate dehydratase gene; ARO10: phenylpyruvate decarboxylase gene from S. cerevisiae; feaB: phenylacetaldehyde dehydrogenase gene. **Figure S2**. CRISPRi-based gene repression. (A) Block transcription initiation via competition with RNA polymerase (RNAP) or transcription factors (TFs) for binding to promoter DNA. When the dCas9-sgRNA complex binds to the promoter sequence or the cis-acting transcription factor binding site (TFBS), it can block transcription initiation by sterically inhibiting the binding of RNAP or transcription factors (TFs). Silencing of transcription initiation is effective for both nontemplate (NT) and template (T) DNA strand. (B) Block transcription elongation by targeting the CDS and preventing the transcription elongation complex from passing. When the dCas9-sgRNA complex binds to the NT DNA strand of the UTR or the protein coding region, it can repress gene expression by blocking the elongating RNAPs. Silencing of transcription elongation is effective only for the NT DNA strand. Additionally, the interference is negatively correlated with the distance of the target site from the transcription start site. Thus, to achieve better repression, target sites within the 5’ end of the gene should be selected. **Figure S3.** Effect of repression of NADHP-consuming enzyme-encoding genes on the production of 4HPAA. **Figure S4.** Effect of repression of ATP-consuming enzyme-encoding genes on the production of 4HPAA. **Figure S5**. (A) The 4HPAA tolerance of E. coli 4HPAA (■) and E. coli 4HPAA-GS-2-4 (●). Cells were cultured in the presence of 35.0 g/L 4HPAA at 30°C and 200 rpm for 24 h. (B) The 4HPAA production (gray bar) and growth (white bar) of E. coli 4HPAA and E. coli 4HPAA-GS-2-4. **Figure S6**. Plasmid maps used for CRISPRi in this study. **Table S1**. Transcriptional levels of the selected genes in E. coli 4HPAA-2 after CRISPRi-based repression compared with those without sgRNAs. **Table S2.** Primers used in this study.

## Data Availability

Not applicable.
