# Supplementary material for: ATP and NADPH engineering of Escherichia coli to improve the production of 4-hydroxyphenylacetic acid using CRISPRi
Source: Biotechnol Biofuels. 2021 Apr 20;14:100. doi: 10.1186/s13068-021-01954-6 (PMC8056492; doi:10.1186/s13068-021-01954-6)
Supplement: Supplementary file 1 — Additional file 1: Figure S1 Biosynthetic pathway of 4-hydroxyphenylacetic acid in the engineered Escherichia coli used in this study. Black: E. coli genes; red: Saccharomyces cerevisiae gene. PEP: phosphoenolpyruvate; E4P: D-erythrose 4-phosphate; DAHP: 3-deoxy-arabino-heptulonate 7-phosphate; DHQ: 3-dehydroquinate; DHS: 3-dehydroshikimate; EPSP: enolpyruvoyl-shikimate 3-phosphate. aroF/aroH/aroG: 3-deoxy-7-phosphoheptulonate synthase gene; aroB: 3-dehydroquinate synthase gene; aroD: 3-dehydroquinate dehydratase gene; aroE: shikimate dehydrogenase gene; aroK/aroL: shikimate kinase gene; aroA: 3-phosphoshikimate 1-carboxyvinyltransferase gene; aroC: chorismate synthase gene; tyrA: chorismate mutase/prephenate dehydrogenase gene; pheA: chorismate mutase/prephenate dehydratase gene; ARO10: phenylpyruvate decarboxylase gene from S. cerevisiae; feaB: phenylacetaldehyde dehydrogenase gene. Figure S2. CRISPRi-based gene repression. (A) Block transcription initiation via competition with RNA polymerase (RNAP) or transcription factors (TFs) for binding to promoter DNA. When the dCas9-sgRNA complex binds to the promoter sequence or the cis-acting transcription factor binding site (TFBS), it can block transcription initiation by sterically inhibiting the binding of RNAP or transcription factors (TFs). Silencing of transcription initiation is effective for both nontemplate (NT) and template (T) DNA strand. (B) Block transcription elongation by targeting the CDS and preventing the transcription elongation complex from passing. When the dCas9-sgRNA complex binds to the NT DNA strand of the UTR or the protein coding region, it can repress gene expression by blocking the elongating RNAPs. Silencing of transcription elongation is effective only for the NT DNA strand. Additionally, the interference is negatively correlated with the distance of the target site from the transcription start site. Thus, to achieve better repression, target sites within the 5’ end of the gene should be s [file 13068_2021_1954_MOESM1_ESM.docx]

**ATP and NADPH engineering of *Escherichia coli* to improve the production of 4-hydroxyphenylacetic acid using CRISPRi**

*Yu-Ping Shen^1,2^, Yu-Ling Liao^1^, Qian Lu^1^, Xin He^1^, Zhi-Bo Yan^1^, Jian-Zhong Liu^1^**

1. *Institute of Synthetic Biology, Biomedical Center,* *Guangdong Province Key Laboratory of* *Improved Variety Reproduction in Aquatic Economic Animals, School of Life Sciences,* *Sun Yat-sen University, Guangzhou 510275, China*
2. *College of Chemistry and Bioengineering, Hunan University of Science and Engineering, Yongzhou 425199, China*

Email:

Yu-Ping Shen: shenyup@mail2.sysu.edu.cn

Yu-Ling Liao: liaoyling@mail.sysu.edu.cn

*Qian Lu:* *luqian5@mail2.sysu.edu.cn*

*Xin He: hexin37@mail2.sysu.edu.cn*

Zhi-Bo Yan: yanzhb3@mail2.sysu.edu.cn

Jian-Zhong Liu: lssljz@mail.sysu.edu.cn

*Corresponding author: Institute of Synthetic Biology, School of Life Science, Sun Yat-Sen University, Guangzhou 510275, P.R. China. Phone: +86-20-84110115. Fax: +86-20-84036461. *E-mail address*: lssljz@mail.sysu.edu.cn (J. Z. Liu)

**Supplementary Figure 1 Biosynthetic pathway of 4-hydroxyphenylacetic acid in the engineered *Escherichia coli* used in this study.** Black: *E. coli* genes; Red: *Saccharomyces cerevisiae* gene. PEP: phosphoenolpyruvate; E4P: D-erythrose 4-phosphate; DAHP: 3-deoxy-arabino-heptulonate 7-phosphate; DHQ: 3-dehydroquinate; DHS: 3-dehydroshikimate; EPSP: enolpyruvoyl-shikimate 3-phosphate. *aroF/aroH/aroG*: 3-deoxy-7-phosphoheptulonate synthase gene; *aroB*: 3-dehydroquinate synthase gene; *aroD:* 3-dehydroquinate dehydatase gene; *aroE*: shikimate dehydrogenase gene; *aroK/aroL*: shikimate kinase gene; *aroA:* 3-phosphoshikimate 1-carboxyvinyltransferase gene; *aroC:* chorismate synthase gene; *tyrA:* chorismate mutase/prephenate dehydrogenase gene; *pheA:* chorismate mutase/prephenate dehydratase gene; *ARO10:* phenylpyruvate decarboxylase gene from *S. cerevisiae*; *feaB*: phenylacetaldehyde dehydrogenase gene.


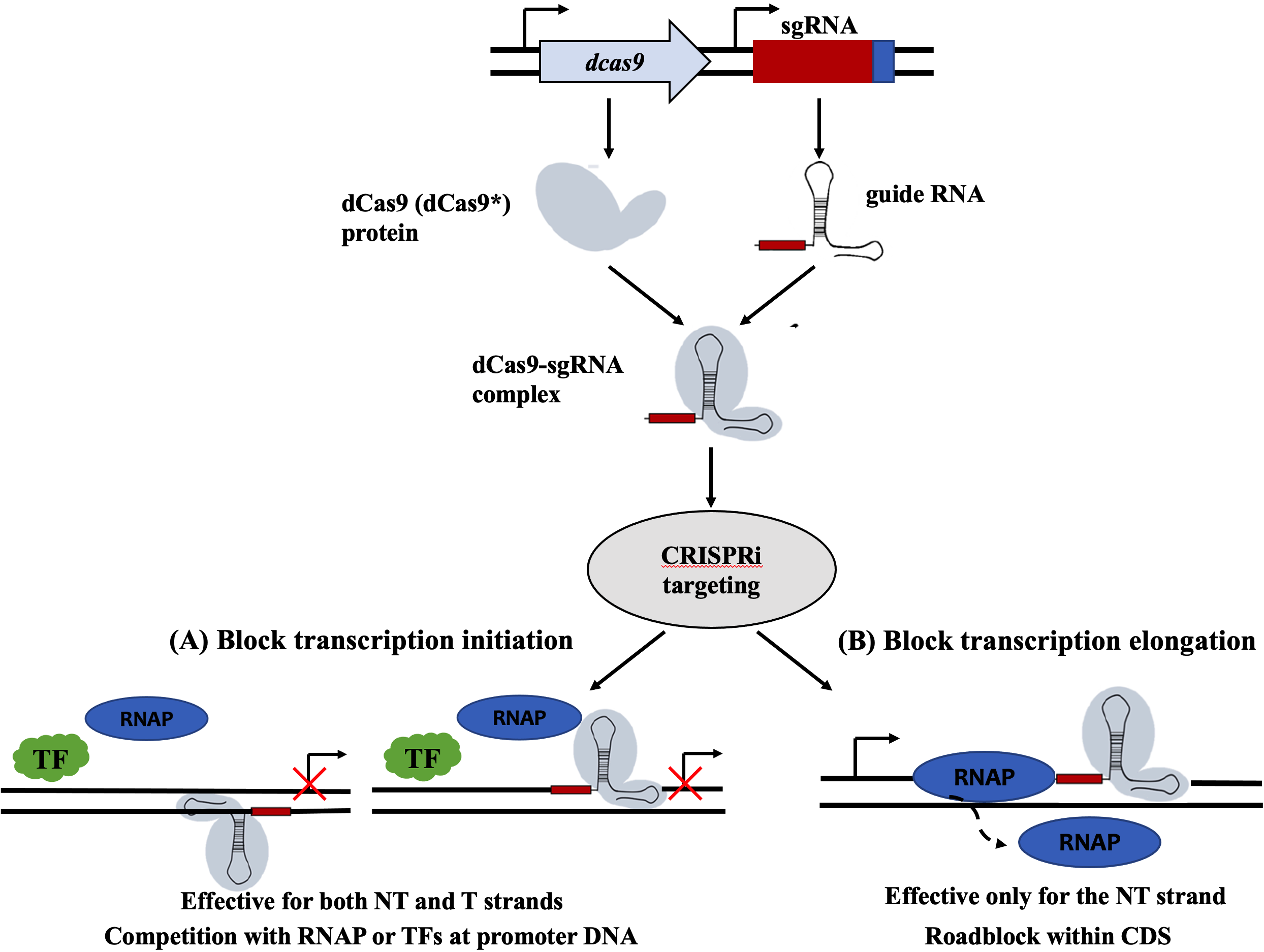


**Supplementary Figure 2 CRISPRi-based gene repression.** (A) Block transcription initiation via competition with RNA polymerase (RNAP) or transcription factors (TFs) for binding to promoter DNA. When the dCas9-sgRNA complex binds to the promoter sequence or the *cis*-acting transcription factor binding site (TFBS), it can block transcription initiation by sterically inhibiting the binding of RNAP or transcription factors (TFs). Silencing of transcription initiation is effective for both nontemplate (NT) and template (T) DNA strand. (B) Block transcription elongation by targeting the CDS and preventing the transcription elongation complex from passing. When the dCas9-sgRNA complex binds to the NT DNA strand of the UTR or the protein coding region, it can repress gene expression by blocking the elongating RNAPs. Silencing of transcription elongation is effective only for the NT DNA strand. Additionally, the interference is negatively correlated with the distance of the target site from the transcription start site. Thus, to achieve better repression, target sites within the 5’ end of the gene should be selected.

**Supplementary Figure 3 Effect of repression of NADHP-consuming enzyme-encoding genes on the production of 4HPAA**

**Supplementary Figure 4 Effect of repression of ATP-consuming enzyme-encoding genes on the production of 4HPAA**

**Supplementary Figure 5 (A) The 4HPAA tolerance of *E. coli* 4HPAA (◼) and *E. coli* 4HPAA-GS-2-4 (⚫).** Cells were cultured in the presence of 35.0 g/L 4HPAA at 30°C and 200 rpm for 24 h. (B) The 4HPAA production (gray bar) and growth (white bar) of *E. coli* 4HPAA and *E. coli* 4HPAA-GS-2-4.

**Supplementary Table 1 Transcriptional levels of the selected genes in *E. coli* 4HPAA-2 after CRISPRi-based repression comparted with those without sgRNAs.** *The Ratio of transcriptional level with CRISPRi and without. The data represent the means of three replicates, and error bars represent standard deviations.

| Gene | Protein | Ratio* |
| --- | --- | --- |
| *araH* | arabinose ABC transporter membrane subunit, ATP-consuming | 0.26±0.00 |
| *fecE* | ferric citrate ABC transporter ATP binding subunit, ATP-consuming | 0.20±0.09 |
| *yahK* | aldehyde reductase, NADPH-dependent | 0.37±0.01 |
| *yqjH* | ferric reductase, NADPH-dependent | 0.28±0.06 |

## Quantitative real-time PCR (qRT-PCR)

The total RNA from *E. coli* cells grown for 38 h in shake flasks was isolated using an RNA extraction kit (Dongsheng Biotech, Guangzhou, China) following the manufacturer’s instructions. The first-strand cDNA was synthesized using an All-in-OneTM First-Strand cDNA Synthesis kit (GeneCopoeia, Guangzhou, China). Quantitative real-time PCR was performed with the All-in-OneTM qPCR Mix kit (GeneCopoeia) by an iCycler iQ5 Real Time PCR system (Bio-Rad Laboratories, California, USA). The template was 100 ng of cDNA. The PCR conditions were as follows: 95 °C for 10 min, followed by 45 cycles of denaturation at 95 °C for 10 s, annealing at 60 °C for 20 s, and extension at 72 °C for 15 s. The primers for qRT-PCR are presented in Supplementary Table 1. The expression levels were analyzed by the 2^-△△Ct^ method described by Livak and Schmittgen [1] and normalized by *cysG* gene expression. Three biological replicates for each sample were used for qRT-PCR analysis, and three technical replicates were analyzed for each biological replicate.

**Supplementary Table 2 Primers used in this study**

| Name | | 5'-3' | | Purpose | |
| --- | --- | --- | --- | --- | --- |
| **N20 for NADPH-consuming enzyme-encoding genes** | | | | | |
| Target-ahr-F | | ATACTAGTTTCGCCGCCCGCTTCTTTTGGTTTTAGAGCTAGAAATAGCAAG | | anti-ahr | |
| Target-aldB-F | | ATACTAGTCTGTGCTGAAGGGGGATTATGTTTTAGAGCTAGAAATAGCAAG | | anti-aldB | |
| Target-argC-F | | ATACTAGTCTCTGCGCCAGCGTAGCCGCGTTTTAGAGCTAGAAATAGCAAG | | anti-argC | |
| Target-aroE-F | | ATACTAGTCGGATTACCAAAAACAGCATGTTTTAGAGCTAGAAATAGCAAG | | anti-aroE | |
| Target-asd-F | | ATACTAGTAACCATGCGTTGCATGAGAAGTTTTAGAGCTAGAAATAGCAAG | | anti-asd | |
| Target-curA-F | | ATACTAGTAGGTGCGCCATGTGGACGCGGTTTTAGAGCTAGAAATAGCAAG | | anti-curA | |
| Target-cysI-F | | ATACTAGTCCTTCGACCACTAAAGGCCCGTTTTAGAGCTAGAAATAGCAAG | | anti-cysI | |
| Target-cysJ-F | | ATACTAGTACGGAAGCAACGCGGAAGGTGTTTTAGAGCTAGAAATAGCAAG | | anti-cysJ | |
| Target-dapB-F | | ATACTAGTACGCCCCCCGGCTCCCGCGAGTTTTAGAGCTAGAAATAGCAAG | | anti-dapB | |
| Target-dkgA-F | | ATACTAGTTCCTGTAGCTTAATAACGGTGTTTTAGAGCTAGAAATAGCAAG | | anti-dkgA | |
| Target-dkgB-F | | ATACTAGTAAAGTACCTAAACCAAATGCGTTTTAGAGCTAGAAATAGCAAG | | anti-dkgB | |
| Target-dusA-F | | ATACTAGTAAAGCAGACGCAAGAAATAGGTTTTAGAGCTAGAAATAGCAAG | | anti-dusA | |
| Target-dusB-F | | ATACTAGTCAGCCATGGGCGCTGCGATCGTTTTAGAGCTAGAAATAGCAAG | | anti-dusB | |
| Target-dusC-F | | ATACTAGTGAGTCAAGCACTCCCTCCATGTTTTAGAGCTAGAAATAGCAAG | | anti-dusC | |
| Target-dxr-F | | ATACTAGTCGAGCCGGTCGAGCCCAGAAGTTTTAGAGCTAGAAATAGCAAG | | anti-dxr | |
| Target-epmC-F | | ATACTAGTCAGACGGGTATTAAAATCATGTTTTAGAGCTAGAAATAGCAAG | | anti-epmC | |
| Target-fabG-F | | ATACTAGTCAATTGCGCGGCCAATTCCGGTTTTAGAGCTAGAAATAGCAAG | | anti-fabG | |
| Target-fadH-F | | ATACTAGTTCCAGCGGGGCGAACAGCGAGTTTTAGAGCTAGAAATAGCAAG | | anti-fadH | |
| Target-fcl-F | | ATACTAGTTTCGAGCTGCCGCCTGATGGGTTTTAGAGCTAGAAATAGCAAG | | anti-fcl | |
| Target-folA-F | | ATACTAGTAGATCGGCAGGCAGGTTCCAGTTTTAGAGCTAGAAATAGCAAG | | anti-folA | |
| Target-folD-F | | ATACTAGTCAGTCCTGGTGCCCGCAGTCGTTTTAGAGCTAGAAATAGCAAG | | anti-folD | |
| Target-folM-F | | ATACTAGTAATTAATATTGGCAAGGGCTGTTTTAGAGCTAGAAATAGCAAG | | anti-folM | |
| Target-fpr-F | | ATACTAGTGGTGAGACTAAACAGGGCGTGTTTTAGAGCTAGAAATAGCAAG | | anti-fpr | |
| Target-fre-F | | ATACTAGTCGAGGTCACTTTACAGCTTAGTTTTAGAGCTAGAAATAGCAAG | | anti-fre | |
| Target-gabD-F | | ATACTAGTCGTTAATCAACGCCTGCTGGGTTTTAGAGCTAGAAATAGCAAG | | anti-gabD | |
| Target-gdhA-F | | ATACTAGTCGCGCTTTTGGACATGGTTGGTTTTAGAGCTAGAAATAGCAAG | | anti-gdhA | |
| Target-ghrA-F | | ATACTAGTACCATTGGGTATCGAACGTTGTTTTAGAGCTAGAAATAGCAAG | | anti-ghrA | |
| Target-ghrB-F | | ATACTAGTGCTTTGTAGAGGATAACGGAGTTTTAGAGCTAGAAATAGCAAG | | anti-ghrB | |
| Target-gltD-F | | ATACTAGTATCTTCAGCGGTTTCTTTGGGTTTTAGAGCTAGAAATAGCAAG | | anti-gltD | |
| Target-gltB-F | | ATACTAGTACCACAGTTATCCCTCTCAAGTTTTAGAGCTAGAAATAGCAAG | | anti-gltB | |
| Target-gnd-F | | ATACTAGTGACTACGCCGATCTGTTGCTGTTTTAGAGCTAGAAATAGCAAG | | anti-gnd | |
| Target-gor-F | | ATACTAGTGTACATAGCCGCGCGGTTGAGTTTTAGAGCTAGAAATAGCAAG | | anti-gor | |
| Target-gpr-F | | ATACTAGTTGCATCTGCCCGTAACGTTCGTTTTAGAGCTAGAAATAGCAAG | | anti-gpr | |
| Target-guaC-F | | ATACTAGTTAAGAGTGGAGCGTTTAGGGGTTTTAGAGCTAGAAATAGCAAG | | anti-guaC | |
| Target-hcxB-F | | ATACTAGTGTTTGCCGCGATTAAATGATGTTTTAGAGCTAGAAATAGCAAG | | anti-hcxB | |
| Target-hemA-F | | ATACTAGTGTTGATACCGAGTGCTAAAAGTTTTAGAGCTAGAAATAGCAAG | | anti-hemA | |
| Target-icd-F | | ATACTAGTGTGATCTTCTTGCCTTGTGCGTTTTAGAGCTAGAAATAGCAAG | | anti-icd | |
| Target-ilvC-F | | ATACTAGTCGCCATCGGCGAATTCATCGGTTTTAGAGCTAGAAATAGCAAG | | anti-ilvC | |
| Target-mdaB-F | | ATACTAGTTTTTCGCGCCGTTGATAATCGTTTTAGAGCTAGAAATAGCAAG | | anti-mdaB | |
| Target-murB-F | | ATACTAGTTGTTCCAGGGTTTTAAGGAGGTTTTAGAGCTAGAAATAGCAAG | | anti-murB | |
| Target-nemA-F | | ATACTAGTGATCGCGCCCACTTTCAGTGGTTTTAGAGCTAGAAATAGCAAG | | anti-nemA | |
| Target-nfsA-F | | ATACTAGTCCACAAATAAGTTCAATGGTGTTTTAGAGCTAGAAATAGCAAG | | anti-nfsA | |
| Target-paaB-F | | ATACTAGTCGCACGAAAACTTCGTATAAGTTTTAGAGCTAGAAATAGCAAG | | anti-paaB | |
| Target-paaC-F | | ATACTAGTGCAGTTATCGCCCAGGCGCAGTTTTAGAGCTAGAAATAGCAAG | | anti-paaC | |
| Target-paaA-F | | ATACTAGTTTGCTCAAAGCGTTCTTCTTGTTTTAGAGCTAGAAATAGCAAG | | anti-paaA | |
| Target-paaE-F | | ATACTAGTCTCGACTTTTGCCACTGTTAGTTTTAGAGCTAGAAATAGCAAG | | anti-paaE | |
| Target-paaZ-F | | ATACTAGTCCGGCCAGACTGCCAGGTACGTTTTAGAGCTAGAAATAGCAAG | | anti-paaZ | |
| Target-panE-F | | ATACTAGTTAAGGCACCGCATCCCAATAGTTTTAGAGCTAGAAATAGCAAG | | anti-panE | |
| Target-pdxI-F | | ATACTAGTGCCATAACCAAGACGGTTAAGTTTTAGAGCTAGAAATAGCAAG | | anti-pdxI | |
| Target-pntA-F | | ATACTAGTTCATTGGTTAACCGTTCTCTGTTTTAGAGCTAGAAATAGCAAG | | anti-pntA | |
| Target-pntB-F | | ATACTAGTACTGAAGATAAACAGGATCGGTTTTAGAGCTAGAAATAGCAAG | | anti-pntB | |
| Target-proA-F | | ATACTAGTTTTATACGAGGCTTGCTTCGGTTTTAGAGCTAGAAATAGCAAG | | anti-proA | |
| Target-qorA-F | | ATACTAGTCTACGGCTTGAAGTACTTCCGTTTTAGAGCTAGAAATAGCAAG | | anti-qorA | |
| Target-qorB-F | | ATACTAGTATAGTGACCAAGTTGGCCAGGTTTTAGAGCTAGAAATAGCAAG | | anti-qorB | |
| Target-queF-F | | ATACTAGTTCAGGCCCGCAAGTGCCTGAGTTTTAGAGCTAGAAATAGCAAG | | anti-queF | |
| Target-rfbD-F | | ATACTAGTGCCCTGTTTTGCCAAAAAGGGTTTTAGAGCTAGAAATAGCAAG | | anti-rfbD | |
| Target-ribD-F | | ATACTAGTGACATTCGGGTTGGGATGCGGTTTTAGAGCTAGAAATAGCAAG | | anti-ribD | |
| Target-rutE-F | | ATACTAGTGCTAAGCGCACCTGGGCTAAGTTTTAGAGCTAGAAATAGCAAG | | anti-rutE | |
| Target-ssuE-F | | ATACTAGTAAAGCGAGGACTACCCGCCAGTTTTAGAGCTAGAAATAGCAAG | | anti-ssuE | |
| Target-sthA-F | | ATACTAGTGCATCGTAATCGTAGGAATGGTTTTAGAGCTAGAAATAGCAAG | | anti-sthA | |
| Target-thiH-F | | ATACTAGTTTGTCGCCAGCGATCGCTGAGTTTTAGAGCTAGAAATAGCAAG | | anti-thiH | |
| Target-trxB-F | | ATACTAGTGATAAGCAGTTTACTGTGTTGTTTTAGAGCTAGAAATAGCAAG | | anti-trxB | |
| Target-ubiH-F | | ATACTAGTCAGCGCCCCGTGACTTAACCGTTTTAGAGCTAGAAATAGCAAG | | anti-ubiH | |
| Target-ubiI-F | | ATACTAGTCACCATGCCGCCGCCAACAAGTTTTAGAGCTAGAAATAGCAAG | | anti-ubiI | |
| Target-yahK-F | | ATACTAGTCGGTTCAAGTGGTTGTTTAGGTTTTAGAGCTAGAAATAGCAAG | | anti-yahK | |
| Target-ybbO-F | | ATACTAGTTTTGCATAACTTTACCTGTCGTTTTAGAGCTAGAAATAGCAAG | | anti-ybbO | |
| Target-ydfG-F | | ATACTAGTCCGTTCCTGGCGACGGCCAGGTTTTAGAGCTAGAAATAGCAAG | | anti-ydfG | |
| Target-yeaE-F | | ATACTAGTATACCATGTTCCCTGCCCTAGTTTTAGAGCTAGAAATAGCAAG | | anti-yeaE | |
| Target-yghA-F | | ATACTAGTCAGTGTAATACTGCGTGGTCGTTTTAGAGCTAGAAATAGCAAG | | anti-yghA | |
| Target-yhdH-F | | ATACTAGTCCGTGACATCGCCCTCCGGCGTTTTAGAGCTAGAAATAGCAAG | | anti-yhdH | |
| Target-yjeF-F | | ATACTAGTCGGGGTTTTTCTTCATTGTAGTTTTAGAGCTAGAAATAGCAAG | | anti-yjeF | |
| Target-yohF-F | | ATACTAGTGCCGATCCCCGAATCGGAGGGTTTTAGAGCTAGAAATAGCAAG | | anti-yohF | |
| Target-yqhD-F | | ATACTAGTTTACCAAACAGAATGCGGGTGTTTTAGAGCTAGAAATAGCAAG | | anti-yqhD | |
| Target-yqjH-F | | ATACTAGTAACGCGCTGCGGGTAGCGGGGTTTTAGAGCTAGAAATAGCAAG | | anti-yqjH | |
| Target-zwf-F | | ATACTAGTAATGACCAGGTCACAGGCCTGTTTTAGAGCTAGAAATAGCAAG | | anti-zwf | |
| **N20 for NADPH-consuming enzyme-encoding genes** | | | | | |
| Target-aas-F | | ATACTAGTCGCGAACGCGATACAAAACAGTTTTAGAGCTAGAAATAGCAAG | | anti-aas | |
| Target-accC-F | | ATACTAGTCAATGCAATCTCGCCGCGGTGTTTTAGAGCTAGAAATAGCAAG | | anti-accC | |
| Target-aceK-F | | ATACTAGTATCAATAATTCCAGGCCACGGTTTTAGAGCTAGAAATAGCAAG | | anti-aceK | |
| Target-ackA-F | | ATACTAGTACCATTTACTGCATCGATGAGTTTTAGAGCTAGAAATAGCAAG | | anti-ackA | |
| Target-acs-F | | ATACTAGTGCTGAGGGTTTATCAGGCAAGTTTTAGAGCTAGAAATAGCAAG | | anti-acs | |
| Target-adk-F | | ATACTAGTTGAGTCCCTTTCCCCGCGCCGTTTTAGAGCTAGAAATAGCAAG | | anti-adk | |
| Target-alaS-F | | ATACTAGTAAACGCCTGACGGATCTCAGGTTTTAGAGCTAGAAATAGCAAG | | anti-alaS | |
| Target-alsB-F | | ATACTAGTCAATACGACAGCATATTCGGGTTTTAGAGCTAGAAATAGCAAG | | anti-alsB | |
| Target-alsC-F | | ATACTAGTCAGAACAGCGCAAAGTTGAAGTTTTAGAGCTAGAAATAGCAAG | | anti-alsC | |
| Target-alsA-F | | ATACTAGTATCCCCGCCATCGATATATAGTTTTAGAGCTAGAAATAGCAAG | | anti-alsA | |
| Target-alsK-F | | ATACTAGTCTGCTGTCCGCAGACAAAAGGTTTTAGAGCTAGAAATAGCAAG | | anti-alsK | |
| Target-anmK-F | | ATACTAGTCTGACATAACGCCAATAAAGGTTTTAGAGCTAGAAATAGCAAG | | anti-anmK | |
| Target-araB-F | | ATACTAGTCAGAATCACTGCCAAAATCGGTTTTAGAGCTAGAAATAGCAAG | | anti-araB | |
| Target-araF-F | | ATACTAGTTGCCAGACCAATGGCTGCCAGTTTTAGAGCTAGAAATAGCAAG | | anti-araF | |
| Target-araH-F | | ATACTAGTCCGAAGCTGAATGACGACTTGTTTTAGAGCTAGAAATAGCAAG | | anti-araH | |
| Target-araG-F | | ATACTAGTGCCGCGAAATGAGAGATACGGTTTTAGAGCTAGAAATAGCAAG | | anti-araG | |
| Target-argB-F | | ATACTAGTCCGCCCAGTTTGATAATTAAGTTTTAGAGCTAGAAATAGCAAG | | anti-argB | |
| Target-argG-F | | ATACTAGTTACCAATACGTTGACCTACCGTTTTAGAGCTAGAAATAGCAAG | | anti-argG | |
| Target-argS-F | | ATACTAGTCTGCCGCAATCATGGCCTGAGTTTTAGAGCTAGAAATAGCAAG | | anti-argS | |
| Target-argT-F | | ATACTAGTCGCATAGCTGGAAGCCGCTGGTTTTAGAGCTAGAAATAGCAAG | | anti-argT | |
| Target-hisM-F | | ATACTAGTTAACCGTCGGTCCACAGCAGGTTTTAGAGCTAGAAATAGCAAG | | anti-hisM | |
| Target-hisP-F | | ATACTAGTGATAACGTTTAATTTATTCTGTTTTAGAGCTAGAAATAGCAAG | | anti-hisP | |
| Target-hisQ-F | | ATACTAGTCGGCACGCCACGAATCAGCGGTTTTAGAGCTAGAAATAGCAAG | | anti-hisQ | |
| Target-aroK-F | | ATACTAGTGTGCTTTTTCCGGCACCCATGTTTTAGAGCTAGAAATAGCAAG | | anti-aroK | |
| Target-aroL-F | | ATACTAGTCGAGGCCCGATCAGAAAAAGGTTTTAGAGCTAGAAATAGCAAG | | anti-aroL | |
| Target-artP-F | | ATACTAGTACACCAGCGTTTCGCCCTGTGTTTTAGAGCTAGAAATAGCAAG | | anti-artP | |
| Target-artM-F | | ATACTAGTTGTAGCCCTTTCATCAGTTCGTTTTAGAGCTAGAAATAGCAAG | | anti-artM | |
| Target-artQ-F | | ATACTAGTAAGGCCGACGGTCATCCCGGGTTTTAGAGCTAGAAATAGCAAG | | anti-artQ | |
| Target-artJ-F | | ATACTAGTAGTAAAGGAAGCAAGTAAAGGTTTTAGAGCTAGAAATAGCAAG | | anti-artJ | |
| Target-asnA-F | | ATACTAGTACGTTGTTTGGCAATGTAAGGTTTTAGAGCTAGAAATAGCAAG | | anti-asnA | |
| Target-asnB-F | | ATACTAGTCATCAGGCGTGACAGCTCGAGTTTTAGAGCTAGAAATAGCAAG | | anti-asnB | |
| Target-asnS-F | | ATACTAGTCCCTGGAGTACGTCGGCTACGTTTTAGAGCTAGAAATAGCAAG | | anti-asnS | |
| Target-aspS-F | | ATACTAGTGCTGCCCCACGTGGGACAAAGTTTTAGAGCTAGAAATAGCAAG | | anti-aspS | |
| Target-atpC-F | | ATACTAGTCTGCGCTGACGACGTCCAGGGTTTTAGAGCTAGAAATAGCAAG | | anti-atpC | |
| Target-atpH-F | | ATACTAGTCAAAAGCTGCTTTGGCGTAGGTTTTAGAGCTAGAAATAGCAAG | | anti-atpH | |
| Target-atpA-F | | ATACTAGTGATCAGTTCGCTGATTTCGGGTTTTAGAGCTAGAAATAGCAAG | | anti-atpA | |
| Target-atpG-F | | ATACTAGTACTACGTATCTCTTTTGCGCGTTTTAGAGCTAGAAATAGCAAG | | anti-atpG | |
| Target-atpD-F | | ATACTAGTCAACTACGGCGCCGATTACCGTTTTAGAGCTAGAAATAGCAAG | | anti-atpD | |
| Target-atpE-F | | ATACTAGTTGTACAGCAGATCCATATTCGTTTTAGAGCTAGAAATAGCAAG | | anti-atpE | |
| Target-atpF-F | | ATACTAGTCAAACGCGATGGCCTGGCCGGTTTTAGAGCTAGAAATAGCAAG | | anti-atpF | |
| Target-atpB-F | | ATACTAGTTGGTGTCCTATGTAATCCTGGTTTTAGAGCTAGAAATAGCAAG | | anti-atpB | |
| Target-bioD-F | | ATACTAGTCCCCACTTCGGTATCCGTTCGTTTTAGAGCTAGAAATAGCAAG | | anti-bioD | |
| Target-birA-F | | ATACTAGTGGCAATCAATTTCAGTGGCAGTTTTAGAGCTAGAAATAGCAAG | | anti-birA | |
| Target-btuF-F | | ATACTAGTCAGTGGCGCAAGAAAAGACAGTTTTAGAGCTAGAAATAGCAAG | | anti-btuF | |
| Target-btuC-F | | ATACTAGTTATTTTGTCGCTGTTGTTGGGTTTTAGAGCTAGAAATAGCAAG | | anti-btuC | |
| Target-btuD-F | | ATACTAGTCGCCAGAAAGCGGCCCCAGGGTTTTAGAGCTAGAAATAGCAAG | | anti-btuD | |
| Target-btuR-F | | ATACTAGTTCACTCGCTGCTGACGCTGTGTTTTAGAGCTAGAAATAGCAAG | | anti-btuR | |
| Target-caiC-F | | ATACTAGTCCGGTTAACGACTCCGCCGCGTTTTAGAGCTAGAAATAGCAAG | | anti-caiC | |
| Target-carA-F | | ATACTAGTTATGGCCCGACCGTGAAACTGTTTTAGAGCTAGAAATAGCAAG | | anti-carA | |
| Target-carB-F | | ATACTAGTCTTTTTATATCTGTACGTTTGTTTTAGAGCTAGAAATAGCAAG | | anti-carB | |
| Target-cca-F | | ATACTAGTACCCAATCTCTGTCTTTGACGTTTTAGAGCTAGAAATAGCAAG | | anti-cca | |
| Target-ccmC-F | | ATACTAGTAGATTTGATACAGCCGTGGTGTTTTAGAGCTAGAAATAGCAAG | | anti-ccmC | |
| Target-ccmE-F | | ATACTAGTCAATCCACAAGCGGTTTTTAGTTTTAGAGCTAGAAATAGCAAG | | anti-ccmE | |
| Target-ccmA-F | | ATACTAGTCCGCTCACAAAGTAACTCTCGTTTTAGAGCTAGAAATAGCAAG | | anti-ccmA | |
| Target-ccmB-F | | ATACTAGTACGCTACACGCAGCTCAAGAGTTTTAGAGCTAGAAATAGCAAG | | anti-ccmB | |
| Target-ccmD-F | | ATACTAGTATTCCAGGAAGCAAATGCAGGTTTTAGAGCTAGAAATAGCAAG | | anti-ccmD | |
| Target-citC-F | | ATACTAGTTATTTTCTGAACGTTTTACGGTTTTAGAGCTAGAAATAGCAAG | | anti-citC | |
| Target-citG-F | | ATACTAGTTAATGACGTCGCAAGCTTTGGTTTTAGAGCTAGAAATAGCAAG | | anti-citG | |
| Target-cmk-F | | ATACTAGTGCCATCAATGGTAATAACCGGTTTTAGAGCTAGAAATAGCAAG | | anti-cmk | |
| Target-coaA-F | | ATACTAGTTTGCGGTCAAACTGTAGGTAGTTTTAGAGCTAGAAATAGCAAG | | anti-coaA | |
| Target-coaD-F | | ATACTAGTGTAATGGGATCGAAAGTACCGTTTTAGAGCTAGAAATAGCAAG | | anti-coaD | |
| Target-coaE-F | | ATACTAGTACTGCCAATGCCTCCCGTTAGTTTTAGAGCTAGAAATAGCAAG | | anti-coaE | |
| Target-cobU-F | | ATACTAGTCCCCAATAAGCGCCTCTGCGGTTTTAGAGCTAGAAATAGCAAG | | anti-cobU | |
| Target-copA-F | | ATACTAGTAGGACAGGCCGTCCAGGGTCGTTTTAGAGCTAGAAATAGCAAG | | anti-copA | |
| Target-cydC-F | | ATACTAGTCGTTTATACAGTGCCAGATAGTTTTAGAGCTAGAAATAGCAAG | | anti-cydC | |
| Target-cydD-F | | ATACTAGTGCTTTGCTGTTTTAACCAGCGTTTTAGAGCTAGAAATAGCAAG | | anti-cydD | |
| Target-cysA-F | | ATACTAGTACCAAACGACTTCTTAATATGTTTTAGAGCTAGAAATAGCAAG | | anti-cysA | |
| Target-cysU-F | | ATACTAGTGCCCGGCAGCACGCGTCTGGGTTTTAGAGCTAGAAATAGCAAG | | anti-cysU | |
| Target-cysW-F | | ATACTAGTCGCGTCATAACGCTTCAATTGTTTTAGAGCTAGAAATAGCAAG | | anti-cysW | |
| Target-sbp-F | | ATACTAGTAATATCCTTTGCCATAACGCGTTTTAGAGCTAGAAATAGCAAG | | anti-sbp | |
| Target-cysC-F | | ATACTAGTGTTGTTGCACAGTGACCGGAGTTTTAGAGCTAGAAATAGCAAG | | anti-cysC | |
| Target-cysN-F | | ATACTAGTGGCGATTTGTTGTGCAAGTGGTTTTAGAGCTAGAAATAGCAAG | | anti-cysN | |
| Target-cysD-F | | ATACTAGTTTTCCGCCTCCAGTTGCCGCGTTTTAGAGCTAGAAATAGCAAG | | anti-cysD | |
| Target-cysP-F | | ATACTAGTGTTCTTTTTCAGTAAGTTAAGTTTTAGAGCTAGAAATAGCAAG | | anti-cysP | |
| Target-cysU-F | | ATACTAGTGCCCGGCAGCACGCGTCTGGGTTTTAGAGCTAGAAATAGCAAG | | anti-cysU | |
| Target-cysW-F | | ATACTAGTCGCGTCATAACGCTTCAATTGTTTTAGAGCTAGAAATAGCAAG | | anti-cysW | |
| Target-cysA-F | | ATACTAGTACCAAACGACTTCTTAATATGTTTTAGAGCTAGAAATAGCAAG | | anti-cysA | |
| Target-cysS-F | | ATACTAGTTAGGCTTAAATTCCTCTTTTGTTTTAGAGCTAGAAATAGCAAG | | anti-cysS | |
| Target-dbpA-F | | ATACTAGTAACATTCAGGGTAGAAAAAGGTTTTAGAGCTAGAAATAGCAAG | | anti-dbpA | |
| Target-ddlA-F | | ATACTAGTGTCGAAGCGACTTTTATCAAGTTTTAGAGCTAGAAATAGCAAG | | anti-ddlA | |
| Target-ddlB-F | | ATACTAGTCAGCGGAGGTCCCACCCAACGTTTTAGAGCTAGAAATAGCAAG | | anti-ddlB | |
| Target-dgkA-F | | ATACTAGTGGTGAATCCAGTGGTATTATGTTTTAGAGCTAGAAATAGCAAG | | anti-dgkA | |
| Target-dgoK-F | | ATACTAGTATAAAGCCAGGCGCGCAGATGTTTTAGAGCTAGAAATAGCAAG | | anti-dgoK | |
| Target-dppA-F | | ATACTAGTCAGCATCCCTGACTTTTTCAGTTTTAGAGCTAGAAATAGCAAG | | anti-dppA | |
| Target-dppC-F | | ATACTAGTTGTAACGGGGTCATCGGCACGTTTTAGAGCTAGAAATAGCAAG | | anti-dppC | |
| Target-dppB-F | | ATACTAGTGGATGACGAGTCCCAAACGTGTTTTAGAGCTAGAAATAGCAAG | | anti-dppB | |
| Target-dppF-F | | ATACTAGTCAACAGCGGTTGTTGCAGGGGTTTTAGAGCTAGAAATAGCAAG | | anti-dppF | |
| Target-dppD-F | | ATACTAGTATGCGGTCTACGGCGCGGAAGTTTTAGAGCTAGAAATAGCAAG | | anti-dppD | |
| Target-entE-F | | ATACTAGTTCTTCCGGCCAGCGGGTGAAGTTTTAGAGCTAGAAATAGCAAG | | anti-entE | |
| Target-entF-F | | ATACTAGTCGGCGACCAAAGGTAAATGCGTTTTAGAGCTAGAAATAGCAAG | | anti-entF | |
| Target-entD-F | | ATACTAGTATGTCCGGCAAAGGGGAGGGGTTTTAGAGCTAGAAATAGCAAG | | anti-entD | |
| Target-entB-F | | ATACTAGTAGTGCGTAAGCCTGTAATTTGTTTTAGAGCTAGAAATAGCAAG | | anti-entB | |
| Target-entF-F | | ATACTAGTCGGCGACCAAAGGTAAATGCGTTTTAGAGCTAGAAATAGCAAG | | anti-entF | |
| Target-epmA-F | | ATACTAGTAAGTTAGGAATGGATGCGCTGTTTTAGAGCTAGAAATAGCAAG | | anti-epmA | |
| Target-etk-F | | ATACTAGTTTTTCCTGAGTGCTGCCTGGGTTTTAGAGCTAGAAATAGCAAG | | anti-etk | |
| Target-fadD-F | | ATACTAGTTCGGAACGTCCGCGGGATAAGTTTTAGAGCTAGAAATAGCAAG | | anti-fadD | |
| Target-fadK-F | | ATACTAGTAGCGGTCTGCTGCCAGTAATGTTTTAGAGCTAGAAATAGCAAG | | anti-fadK | |
| Target-fau-F | | ATACTAGTCCCATTTCCTGCTGTTGTTCGTTTTAGAGCTAGAAATAGCAAG | | anti-fau | |
| Target-fecB-F | | ATACTAGTGCAGGCCTGCAAAAAGAAAAGTTTTAGAGCTAGAAATAGCAAG | | anti-fecB | |
| Target-fecD-F | | ATACTAGTATAACAGCGCACAGCCCGCCGTTTTAGAGCTAGAAATAGCAAG | | anti-fecD | |
| Target-fecC-F | | ATACTAGTCAGCACCGGGTGTTTTATCGGTTTTAGAGCTAGAAATAGCAAG | | anti-fecC | |
| Target-fecE-F | | ATACTAGTAGGGCGGTGATCTTCCCCGTGTTTTAGAGCTAGAAATAGCAAG | | anti-fecE | |
| Target-fepB-F | | ATACTAGTAAGGGCGTTGCGGTAGAGCGGTTTTAGAGCTAGAAATAGCAAG | | anti-fepB | |
| Target-fepG-F | | ATACTAGTGCAAACAGGTGATGAGTAATGTTTTAGAGCTAGAAATAGCAAG | | anti-fepG | |
| Target-fepD-F | | ATACTAGTCACGGCAATGGCGCGTGTCAGTTTTAGAGCTAGAAATAGCAAG | | anti-fepD | |
| Target-fepC-F | | ATACTAGTGCGCAAACGGGCTACTGATTGTTTTAGAGCTAGAAATAGCAAG | | anti-fepC | |
| Target-fhuD-F | | ATACTAGTAGTCGACGGCGCGAAATAAGGTTTTAGAGCTAGAAATAGCAAG | | anti-fhuD | |
| Target-fhuB-F | | ATACTAGTACAGCGCCAGCAATAACGCCGTTTTAGAGCTAGAAATAGCAAG | | anti-fhuB | |
| Target-fhuC-F | | ATACTAGTACGCAGTGCAAAAGTGGTATGTTTTAGAGCTAGAAATAGCAAG | | anti-fhuC | |
| Target-folC-F | | ATACTAGTGCCAGAGGCGACGCGGCTTGGTTTTAGAGCTAGAAATAGCAAG | | anti-folC | |
| Target-folK-F | | ATACTAGTAGAGGCCAGATTGCTGCCTAGTTTTAGAGCTAGAAATAGCAAG | | anti-folK | |
| Target-frlD-F | | ATACTAGTGTTATCGCCGATTGTCGCCAGTTTTAGAGCTAGAAATAGCAAG | | anti-frlD | |
| Target-fruK-F | | ATACTAGTAAGGTCATAAGCCGGATTAAGTTTTAGAGCTAGAAATAGCAAG | | anti-fruK | |
| Target-fucK-F | | ATACTAGTTCGCGCCACAGTCGAGTACCGTTTTAGAGCTAGAAATAGCAAG | | anti-fucK | |
| Target-galK-F | | ATACTAGTGGCAGGGTAGCCAAATGCGTGTTTTAGAGCTAGAAATAGCAAG | | anti-galK | |
| Target-garK-F | | ATACTAGTACTTTCTTTATAAGAGTCTGGTTTTAGAGCTAGAAATAGCAAG | | anti-garK | |
| Target-glgC-F | | ATACTAGTCAACAGATTTCAATGGCAGCGTTTTAGAGCTAGAAATAGCAAG | | anti-glgC | |
| Target-glk-F | | ATACTAGTACACAGAGCAAGACGTGCGTGTTTTAGAGCTAGAAATAGCAAG | | anti-glk | |
| Target-glnA-F | | ATACTAGTCATCGTCAGTACGTGTTCAGGTTTTAGAGCTAGAAATAGCAAG | | anti-glnA | |
| Target-glnE-F | | ATACTAGTTGCTGTAACGGTGAAGAGAGGTTTTAGAGCTAGAAATAGCAAG | | anti-glnE | |
| Target-glnH-F | | ATACTAGTAGAAGAAACCGCAAAAGCCAGTTTTAGAGCTAGAAATAGCAAG | | anti-glnH | |
| Target-glnQ-F | | ATACTAGTCTGGGTTGGGCCAAAGTGCTGTTTTAGAGCTAGAAATAGCAAG | | anti-glnQ | |
| Target-glnS-F | | ATACTAGTACGGATAAAGTTAGTCGGGCGTTTTAGAGCTAGAAATAGCAAG | | anti-glnS | |
| Target-glpK-F | | ATACTAGTCGCGGGAGCTGGTGGTGCCCGTTTTAGAGCTAGAAATAGCAAG | | anti-glpK | |
| Target-gltL-F | | ATACTAGTCCATTTTGAAACATTTTTCAGTTTTAGAGCTAGAAATAGCAAG | | anti-gltL | |
| Target-gltJ-F | | ATACTAGTATAGGTGGTGTTGCCGAACGGTTTTAGAGCTAGAAATAGCAAG | | anti-gltJ | |
| Target-gltK-F | | ATACTAGTATATGGCAGGGAAGGGACAAGTTTTAGAGCTAGAAATAGCAAG | | anti-gltK | |
| Target-gltI-F | | ATACTAGTAGGGCGAGGATTGCTGTGGCGTTTTAGAGCTAGAAATAGCAAG | | anti-gltI | |
| Target-gltX-F | | ATACTAGTTGCAGATAGCCTGTTGGGCTGTTTTAGAGCTAGAAATAGCAAG | | anti-gltX | |
| Target-gluQ-F | | ATACTAGTCTTTGAACAAAAAATAAGGCGTTTTAGAGCTAGAAATAGCAAG | | anti-gluQ | |
| Target-glxK-F | | ATACTAGTAAGCTCTCTTTAAAAGAGTCGTTTTAGAGCTAGAAATAGCAAG | | anti-glxK | |
| Target-glyQ-F | | ATACTAGTGATCAAGCCCTGGAAGGTCCGTTTTAGAGCTAGAAATAGCAAG | | anti-glyQ | |
| Target-glyS-F | | ATACTAGTAGGCTGCGCAGTGCTTTTGGGTTTTAGAGCTAGAAATAGCAAG | | anti-glyS | |
| Target-gmk-F | | ATACTAGTGGATTTACCCGCGCCACTGGGTTTTAGAGCTAGAAATAGCAAG | | anti-gmk | |
| Target-gntK-F | | ATACTAGTAGACGTAAATGTGGTGATCAGTTTTAGAGCTAGAAATAGCAAG | | anti-gntK | |
| Target-gshA-F | | ATACTAGTCCAGCGCCTGTGATACGTCCGTTTTAGAGCTAGAAATAGCAAG | | anti-gshA | |
| Target-gshB-F | | ATACTAGTTGATGTTGATGTTTGCGATGGTTTTAGAGCTAGAAATAGCAAG | | anti-gshB | |
| Target-gsiC-F | | ATACTAGTACCGAGACGATAAACAGCGTGTTTTAGAGCTAGAAATAGCAAG | | anti-gsiC | |
| Target-gsiD-F | | ATACTAGTGTCAGGTTTGACCAGTGGCAGTTTTAGAGCTAGAAATAGCAAG | | anti-gsiD | |
| Target-gsiB-F | | ATACTAGTCCAGCGCCACTAACCCACTAGTTTTAGAGCTAGAAATAGCAAG | | anti-gsiB | |
| Target-gsiA-F | | ATACTAGTGCATCAAGTTCATCACTGTGGTTTTAGAGCTAGAAATAGCAAG | | anti-gsiA | |
| Target-gsk-F | | ATACTAGTTGTTTGGATTTACGTTTACCGTTTTAGAGCTAGAAATAGCAAG | | anti-gsk | |
| Target-gss-F | | ATACTAGTCCCGAACGGGGCATCCTGGCGTTTTAGAGCTAGAAATAGCAAG | | anti-gss | |
| Target-guaA-F | | ATACTAGTGAGAACCGAAGTCCAGAATGGTTTTAGAGCTAGAAATAGCAAG | | anti-guaA | |
| Target-gyrB-F | | ATACTAGTTTTCAGGACTTTGATACTGGGTTTTAGAGCTAGAAATAGCAAG | | anti-gyrB | |
| Target-gyrA-F | | ATACTAGTCCGGTGTAATTTCTCTCGCAGTTTTAGAGCTAGAAATAGCAAG | | anti-gyrA | |
| Target-hipA-F | | ATACTAGTTTCATCCAAGTGACAAGTTTGTTTTAGAGCTAGAAATAGCAAG | | anti-hipA | |
| Target-hisG-F | | ATACTAGTTGAGTCATCACTTAAACGGCGTTTTAGAGCTAGAAATAGCAAG | | anti-hisG | |
| Target-hisJ-F | | ATACTAGTCGCCGCAGTTGCGCTGGAGAGTTTTAGAGCTAGAAATAGCAAG | | anti-hisJ | |
| Target-hisQ-F | | ATACTAGTCGGCACGCCACGAATCAGCGGTTTTAGAGCTAGAAATAGCAAG | | anti-hisQ | |
| Target-hisM-F | | ATACTAGTTAACCGTCGGTCCACAGCAGGTTTTAGAGCTAGAAATAGCAAG | | anti-hisM | |
| Target-hisP-F | | ATACTAGTGATAACGTTTAATTTATTCTGTTTTAGAGCTAGAAATAGCAAG | | anti-hisP | |
| Target-hisS-F | | ATACTAGTGTAATCGTTCATGCCGCGAAGTTTTAGAGCTAGAAATAGCAAG | | anti-hisS | |
| Target-hscA-F | | ATACTAGTAGGTTCACTAATTTGTAATAGTTTTAGAGCTAGAAATAGCAAG | | anti-hscA | |
| Target-hsdS-F | | ATACTAGTGGGGCGATAACCCACCCCTCGTTTTAGAGCTAGAAATAGCAAG | | anti-hsdS | |
| Target-hsdR-F | | ATACTAGTTGAAGTCGTTGACGCCCTTCGTTTTAGAGCTAGAAATAGCAAG | | anti-hsdR | |
| Target-hsdM-F | | ATACTAGTAGGAAACGCCGCCATCGCGCGTTTTAGAGCTAGAAATAGCAAG | | anti-hsdM | |
| Target-hypE-F | | ATACTAGTCGCCGCTACCGTGGGCGAGTGTTTTAGAGCTAGAAATAGCAAG | | anti-hypE | |
| Target-hypF-F | | ATACTAGTCTTTGCCACGAATACGCAGTGTTTTAGAGCTAGAAATAGCAAG | | anti-hypF | |
| Target-idnK-F | | ATACTAGTAAATTTAGCAGATAATAACGGTTTTAGAGCTAGAAATAGCAAG | | anti-idnK | |
| Target-ileS-F | | ATACTAGTCCCTGTTTCCGGCAAATTCAGTTTTAGAGCTAGAAATAGCAAG | | anti-ileS | |
| Target-ispE-F | | ATACTAGTAGATTAAGTTTTGCCGGAGAGTTTTAGAGCTAGAAATAGCAAG | | anti-ispE | |
| Target-kdpC-F | | ATACTAGTAAGATAAATGTTGATAATGCGTTTTAGAGCTAGAAATAGCAAG | | anti-kdpC | |
| Target-kdpB-F | | ATACTAGTAGCGCCTGAACGACAAGTGTGTTTTAGAGCTAGAAATAGCAAG | | anti-kdpB | |
| Target-kdpA-F | | ATACTAGTCATTAACACCAGTAAAAACGGTTTTAGAGCTAGAAATAGCAAG | | anti-kdpA | |
| Target-kdpF-F | | ATACTAGTTAAAAACACCAGCAATACGCGTTTTAGAGCTAGAAATAGCAAG | | anti-kdpF | |
| Target-leuS-F | | ATACTAGTTGGATTCTATCTCTTCCGGGGTTTTAGAGCTAGAAATAGCAAG | | anti-leuS | |
| Target-livF-F | | ATACTAGTGTGGGCGCTGACTTTGTCAAGTTTTAGAGCTAGAAATAGCAAG | | anti-livF | |
| Target-livG-F | | ATACTAGTAGGCCGTTAACAGATAATAAGTTTTAGAGCTAGAAATAGCAAG | | anti-livG | |
| Target-livH-F | | ATACTAGTGCCGATGGCTATCAGCGCGTGTTTTAGAGCTAGAAATAGCAAG | | anti-livH | |
| Target-livM-F | | ATACTAGTAGCGCCATTGCAATATGCATGTTTTAGAGCTAGAAATAGCAAG | | anti-livM | |
| Target-livK-F | | ATACTAGTTTTAATATCGTCAGCCATAGGTTTTAGAGCTAGAAATAGCAAG | | anti-livK | |
| Target-livJ-F | | ATACTAGTACCGTACTGCGCAACCGGACGTTTTAGAGCTAGAAATAGCAAG | | anti-livJ | |
| Target-livM-F | | ATACTAGTAGCGCCATTGCAATATGCATGTTTTAGAGCTAGAAATAGCAAG | | anti-livM | |
| Target-livH-F | | ATACTAGTGCCGATGGCTATCAGCGCGTGTTTTAGAGCTAGAAATAGCAAG | | anti-livH | |
| Target-livG-F | | ATACTAGTAGGCCGTTAACAGATAATAAGTTTTAGAGCTAGAAATAGCAAG | | anti-livG | |
| Target-livF-F | | ATACTAGTGTGGGCGCTGACTTTGTCAAGTTTTAGAGCTAGAAATAGCAAG | | anti-livF | |
| Target-lplA-F | | ATACTAGTAGAGATGAGCAGGCGTAATGGTTTTAGAGCTAGAAATAGCAAG | | anti-lplA | |
| Target-lpxK-F | | ATACTAGTCAGCAATAGCCGCCACAAAGGTTTTAGAGCTAGAAATAGCAAG | | anti-lpxK | |
| Target-lsrA-F | | ATACTAGTGCAAAGTAGCGGTAACGCGCGTTTTAGAGCTAGAAATAGCAAG | | anti-lsrA | |
| Target-lsrC-F | | ATACTAGTCCAGCAGTGCCGTGATTTCAGTTTTAGAGCTAGAAATAGCAAG | | anti-lsrC | |
| Target-lsrD-F | | ATACTAGTAATCTCAATAACGAGCAGTGGTTTTAGAGCTAGAAATAGCAAG | | anti-lsrD | |
| Target-lsrB-F | | ATACTAGTAATGCCAAGAGCGCTAAGTAGTTTTAGAGCTAGAAATAGCAAG | | anti-lsrB | |
| Target-lsrK-F | | ATACTAGTGTAGTACTTTGATTCTGAAAGTTTTAGAGCTAGAAATAGCAAG | | anti-lsrK | |
| Target-lysC-F | | ATACTAGTTACGCTGGTACCGCCAAATTGTTTTAGAGCTAGAAATAGCAAG | | anti-lysC | |
| Target-lysS-F | | ATACTAGTAGGCAATCCCCTGCTCGCGCGTTTTAGAGCTAGAAATAGCAAG | | anti-lysS | |
| Target-lysU-F | | ATACTAGTGTTAAAATCAATAGCCTCATGTTTTAGAGCTAGAAATAGCAAG | | anti-lysU | |
| Target-lyxK-F | | ATACTAGTGCCTTCGCGGTCATACAGCCGTTTTAGAGCTAGAAATAGCAAG | | anti-lyxK | |
| Target-macA-F | | ATACTAGTAATAACGTAACGCTTCTTCAGTTTTAGAGCTAGAAATAGCAAG | | anti-macA | |
| Target-macB-F | | ATACTAGTATATCCTTTAATTCGAGCAAGTTTTAGAGCTAGAAATAGCAAG | | anti-macB | |
| Target-tolC-F | | ATACTAGTGGCTCAGGCCGATAAGAATGGTTTTAGAGCTAGAAATAGCAAG | | anti-tolC | |
| Target-mak-F | | ATACTAGTCAGTGCAATCACTTCAGTTTGTTTTAGAGCTAGAAATAGCAAG | | anti-mak | |
| Target-malE-F | | ATACTAGTTCGTTAATGCGGATAATGCGGTTTTAGAGCTAGAAATAGCAAG | | anti-malE | |
| Target-malG-F | | ATACTAGTGAGCTTTTTGCGATTTCGGTGTTTTAGAGCTAGAAATAGCAAG | | anti-malG | |
| Target-malF-F | | ATACTAGTGTGCGTACATTAAAACAACAGTTTTAGAGCTAGAAATAGCAAG | | anti-malF | |
| Target-malK-F | | ATACTAGTCGATACCACGACCTCGCCCCGTTTTAGAGCTAGAAATAGCAAG | | anti-malK | |
| Target-menE-F | | ATACTAGTACTTGCCGCCAGTGACGCCAGTTTTAGAGCTAGAAATAGCAAG | | anti-menE | |
| Target-metG-F | | ATACTAGTTGGATTGAGCCGTTAGCGTAGTTTTAGAGCTAGAAATAGCAAG | | anti-metG | |
| Target-metK-F | | ATACTAGTAGACGGACTCGGACGTAAAAGTTTTAGAGCTAGAAATAGCAAG | | anti-metK | |
| Target-metL-F | | ATACTAGTGAAACCACCATCATATCGTCGTTTTAGAGCTAGAAATAGCAAG | | anti-metL | |
| Target-metQ-F | | ATACTAGTCAGGGCTCCCACTGCCGCAAGTTTTAGAGCTAGAAATAGCAAG | | anti-metQ | |
| Target-metI-F | | ATACTAGTCGAACCAGCAGCCACATCATGTTTTAGAGCTAGAAATAGCAAG | | anti-metI | |
| Target-metN-F | | ATACTAGTGGTGCCCTGGTGGAACACTTGTTTTAGAGCTAGAAATAGCAAG | | anti-metN | |
| Target-mglB-F | | ATACTAGTGCTGGCCATCACAGCAGACAGTTTTAGAGCTAGAAATAGCAAG | | anti-mglB | |
| Target-mglC-F | | ATACTAGTCGTAAATACCGCCCTCTTTCGTTTTAGAGCTAGAAATAGCAAG | | anti-mglC | |
| Target-mglA-F | | ATACTAGTAACAAGTATTCCCCGGAGGAGTTTTAGAGCTAGAAATAGCAAG | | anti-mglA | |
| Target-mgtA-F | | ATACTAGTAGGTAAATGGCGAATGAGCCGTTTTAGAGCTAGAAATAGCAAG | | anti-mgtA | |
| Target-mnmA-F | | ATACTAGTGCCGACGATTACTTTTTTTGGTTTTAGAGCTAGAAATAGCAAG | | anti-mnmA | |
| Target-modA-F | | ATACTAGTAGCGAAAGAGAGTGCCGCCCGTTTTAGAGCTAGAAATAGCAAG | | anti-modA | |
| Target-modB-F | | ATACTAGTAACTGCCTGCCATTCTGGATGTTTTAGAGCTAGAAATAGCAAG | | anti-modB | |
| Target-modC-F | | ATACTAGTGCAATGGTTGCCCAACGTCTGTTTTAGAGCTAGAAATAGCAAG | | anti-modC | |
| Target-moeB-F | | ATACTAGTAGCCGCGCAGAATGATTTGCGTTTTAGAGCTAGAAATAGCAAG | | anti-moeB | |
| Target-mog-F | | ATACTAGTGCTGGATGCGCGATCAGAGAGTTTTAGAGCTAGAAATAGCAAG | | anti-mog | |
| Target-mpl-F | | ATACTAGTCCGTTACTTCATGGCCTAACGTTTTAGAGCTAGAAATAGCAAG | | anti-mpl | |
| Target-mppA-F | | ATACTAGTTCTGCCAGTACTGTGCCGCTGTTTTAGAGCTAGAAATAGCAAG | | anti-mppA | |
| Target-oppF-F | | ATACTAGTTAAGATCGGCGATTTCAAGGGTTTTAGAGCTAGAAATAGCAAG | | anti-oppF | |
| Target-oppD-F | | ATACTAGTTCAGCCTGTTGTTGTGCGAGGTTTTAGAGCTAGAAATAGCAAG | | anti-oppD | |
| Target-oppC-F | | ATACTAGTTCAGACTGGCAACCGCCGCAGTTTTAGAGCTAGAAATAGCAAG | | anti-oppC | |
| Target-oppB-F | | ATACTAGTGTAATAAGAATAAATAGCGTGTTTTAGAGCTAGAAATAGCAAG | | anti-oppB | |
| Target-msbA-F | | ATACTAGTCAATGGTTGGCCACAGTCGGGTTTTAGAGCTAGAAATAGCAAG | | anti-msbA | |
| Target-murC-F | | ATACTAGTGCGACGCATTTCGGGCACGAGTTTTAGAGCTAGAAATAGCAAG | | anti-murC | |
| Target-murD-F | | ATACTAGTAGGAAAGCCCGGTGAGGCCCGTTTTAGAGCTAGAAATAGCAAG | | anti-murD | |
| Target-murE-F | | ATACTAGTCTGGCACCCACGGAGCAAGAGTTTTAGAGCTAGAAATAGCAAG | | anti-murE | |
| Target-murF-F | | ATACTAGTAATGTCGGTAAGTTGGCTAAGTTTTAGAGCTAGAAATAGCAAG | | anti-murF | |
| Target-nadD-F | | ATACTAGTACCATAGTGCACCGGATCAAGTTTTAGAGCTAGAAATAGCAAG | | anti-nadD | |
| Target-nadE-F | | ATACTAGTTCCTCTTCAGCATTAATCTGGTTTTAGAGCTAGAAATAGCAAG | | anti-nadE | |
| Target-nadK-F | | ATACTAGTTCAGTGCAGTGGGGTGCCGTGTTTTAGAGCTAGAAATAGCAAG | | anti-nadK | |
| Target-nadR-F | | ATACTAGTGTTGCTTGATGGCAGTTTTCGTTTTAGAGCTAGAAATAGCAAG | | anti-nadR | |
| Target-nagK-F | | ATACTAGTTCATAGCTGTCACGCGGTGTGTTTTAGAGCTAGAAATAGCAAG | | anti-nagK | |
| Target-nanK-F | | ATACTAGTGCCGATATCAATCGCCAGTGGTTTTAGAGCTAGAAATAGCAAG | | anti-nanK | |
| Target-ndk-F | | ATACTAGTTACCGCGTTCGGTTTGATGAGTTTTAGAGCTAGAAATAGCAAG | | anti-ndk | |
| Target-nikA-F | | ATACTAGTAAATAGAGTGCGGCGGAGTGGTTTTAGAGCTAGAAATAGCAAG | | anti-nikA | |
| Target-nikC-F | | ATACTAGTCGCCAGGCGTACCGACCAGCGTTTTAGAGCTAGAAATAGCAAG | | anti-nikC | |
| Target-nikB-F | | ATACTAGTTCGGGATCAGCAGCAGAAAGGTTTTAGAGCTAGAAATAGCAAG | | anti-nikB | |
| Target-nikE-F | | ATACTAGTCGCATAGTGATGGGAAAGGCGTTTTAGAGCTAGAAATAGCAAG | | anti-nikE | |
| Target-nikD-F | | ATACTAGTTTACGTAGTTCAATCTGTTGGTTTTAGAGCTAGAAATAGCAAG | | anti-nikD | |
| Target-nrdD-F | | ATACTAGTCCGTCTCGTTTCATCACATGGTTTTAGAGCTAGAAATAGCAAG | | anti-nrdD | |
| Target-oppA-F | | ATACTAGTTAAACTTCTCTTGGTGATGTGTTTTAGAGCTAGAAATAGCAAG | | anti-oppA | |
| Target-paaK-F | | ATACTAGTAATCGGGTCAAGCTTTGTATGTTTTAGAGCTAGAAATAGCAAG | | anti-paaK | |
| Target-panC-F | | ATACTAGTCGAATTTGCTGACGCAGCAGGTTTTAGAGCTAGAAATAGCAAG | | anti-panC | |
| Target-pck-F | | ATACTAGTATAAGCCTCGAGTTCTTGCGGTTTTAGAGCTAGAAATAGCAAG | | anti-pck | |
| Target-pcnB-F | | ATACTAGTGCGGCAAAAATTAGCGACTCGTTTTAGAGCTAGAAATAGCAAG | | anti-pcnB | |
| Target-pdxK-F | | ATACTAGTGTAAACCACCTGCGACTGCAGTTTTAGAGCTAGAAATAGCAAG | | anti-pdxK | |
| Target-pdxY-F | | ATACTAGTGACCATAAACAACGTGAGACGTTTTAGAGCTAGAAATAGCAAG | | anti-pdxY | |
| Target-pfkA-F | | ATACTAGTCGAATTGCGGCGTTCATGCCGTTTTAGAGCTAGAAATAGCAAG | | anti-pfkA | |
| Target-pfkB-F | | ATACTAGTATTGTTGCGCTATCGAGAGAGTTTTAGAGCTAGAAATAGCAAG | | anti-pfkB | |
| Target-pgk-F | | ATACTAGTTTTCCCAGCAAGATCCAGATGTTTTAGAGCTAGAAATAGCAAG | | anti-pgk | |
| Target-pheT-F | | ATACTAGTGCGCATCGCTATCAATCGCCGTTTTAGAGCTAGAAATAGCAAG | | anti-pheT | |
| Target-pheS-F | | ATACTAGTCAGTTCTGCGAGATGTGACAGTTTTAGAGCTAGAAATAGCAAG | | anti-pheS | |
| Target-phnD-F | | ATACTAGTCATGCTGGTGAAGGCCAGCGGTTTTAGAGCTAGAAATAGCAAG | | anti-phnD | |
| Target-phnE_1-F | | ATACTAGTGAACCAGCTGCGCTTGGGTGGTTTTAGAGCTAGAAATAGCAAG | | anti-phnE_1 | |
| Target-phnC-F | | ATACTAGTCAGCGCCTGATGCTGATTGAGTTTTAGAGCTAGAAATAGCAAG | | anti-phnC | |
| Target-phnI-F | | ATACTAGTCGCCTTCTCGCCCCCTTTCAGTTTTAGAGCTAGAAATAGCAAG | | anti-phnI | |
| Target-phnG-F | | ATACTAGTCATCCAGTGCTGGCGGGTCGGTTTTAGAGCTAGAAATAGCAAG | | anti-phnG | |
| Target-phnH-F | | ATACTAGTACTGTGCTGGGCATCCTGCAGTTTTAGAGCTAGAAATAGCAAG | | anti-phnH | |
| Target-phnL-F | | ATACTAGTAGGCGCGATTGAGGACGGGCGTTTTAGAGCTAGAAATAGCAAG | | anti-phnL | |
| Target-phnN-F | | ATACTAGTCAGGCTGTCTTTCCCGGAGCGTTTTAGAGCTAGAAATAGCAAG | | anti-phnN | |
| Target-pncB-F | | ATACTAGTTCCAGCAACGAGTGCAGAACGTTTTAGAGCTAGAAATAGCAAG | | anti-pncB | |
| Target-potD-F | | ATACTAGTGTTGTTGTCATCGGCGTGAGGTTTTAGAGCTAGAAATAGCAAG | | anti-potD | |
| Target-potC-F | | ATACTAGTATACAGGTACGCGTAGATAGGTTTTAGAGCTAGAAATAGCAAG | | anti-potC | |
| Target-potB-F | | ATACTAGTGGTGCCAATGATCATCAGGTGTTTTAGAGCTAGAAATAGCAAG | | anti-potB | |
| Target-potA-F | | ATACTAGTACCAGCGGTGAAAGCGAACTGTTTTAGAGCTAGAAATAGCAAG | | anti-potA | |
| Target-potF-F | | ATACTAGTCGCGAGCGTGCCGACAGAGAGTTTTAGAGCTAGAAATAGCAAG | | anti-potF | |
| Target-potI-F | | ATACTAGTCGCCAGGGCGAACGAACTACGTTTTAGAGCTAGAAATAGCAAG | | anti-potI | |
| Target-potH-F | | ATACTAGTGGCGGTTTCGACTGGGCAGCGTTTTAGAGCTAGAAATAGCAAG | | anti-potH | |
| Target-potG-F | | ATACTAGTTAGCGGCGTCAGCGCCTTACGTTTTAGAGCTAGAAATAGCAAG | | anti-potG | |
| Target-ppk-F | | ATACTAGTAACGCATCCTTTCAATCAGCGTTTTAGAGCTAGAAATAGCAAG | | anti-ppk | |
| Target-ppsA-F | | ATACTAGTCAGCGGTGACGAGCCATTGTGTTTTAGAGCTAGAAATAGCAAG | | anti-ppsA | |
| Target-proB-F | | ATACTAGTCGATCCGCCTGTTAGCACACGTTTTAGAGCTAGAAATAGCAAG | | anti-proB | |
| Target-proS-F | | ATACTAGTTGAGAGTGGAGAGCAGGTATGTTTTAGAGCTAGAAATAGCAAG | | anti-proS | |
| Target-proX-F | | ATACTAGTAGTTTGTGTAGAGATAAGCGGTTTTAGAGCTAGAAATAGCAAG | | anti-proX | |
| Target-proW-F | | ATACTAGTCCAGGCGTCTGCGGATTGCGGTTTTAGAGCTAGAAATAGCAAG | | anti-proW | |
| Target-proV-F | | ATACTAGTATATATTTGAACGCTCGCTGGTTTTAGAGCTAGAAATAGCAAG | | anti-proV | |
| Target-prpE-F | | ATACTAGTTGCTCGGCCCAGAACTGCTCGTTTTAGAGCTAGAAATAGCAAG | | anti-prpE | |
| Target-prs-F | | ATACTAGTCCAGCAAAAAGCTTCATATCGTTTTAGAGCTAGAAATAGCAAG | | anti-prs | |
| Target-pstS-F | | ATACTAGTGGCGACAACAGTTGCGACGGGTTTTAGAGCTAGAAATAGCAAG | | anti-pstS | |
| Target-pstC-F | | ATACTAGTCGGTGGGTTAAAAGCAGGCTGTTTTAGAGCTAGAAATAGCAAG | | anti-pstC | |
| Target-pstA-F | | ATACTAGTAGATTCAGCCAGCGCCGCAGGTTTTAGAGCTAGAAATAGCAAG | | anti-pstA | |
| Target-pstB-F | | ATACTAGTGCCCGATAAACGCCGTTACCGTTTTAGAGCTAGAAATAGCAAG | | anti-pstB | |
| Target-psuK-F | | ATACTAGTATATTGCGCCCTACTCCACCGTTTTAGAGCTAGAAATAGCAAG | | anti-psuK | |
| Target-purC-F | | ATACTAGTCGGGTTTTCCGTGCTGTATAGTTTTAGAGCTAGAAATAGCAAG | | anti-purC | |
| Target-purD-F | | ATACTAGTCGGCGACTGGGCCGCTTTCCGTTTTAGAGCTAGAAATAGCAAG | | anti-purD | |
| Target-purK-F | | ATACTAGTGGCCTAACTGCCCGTTACCGGTTTTAGAGCTAGAAATAGCAAG | | anti-purK | |
| Target-purL-F | | ATACTAGTATTCGGAATGCCGACAGTGCGTTTTAGAGCTAGAAATAGCAAG | | anti-purL | |
| Target-purM-F | | ATACTAGTGGCATCTTTGTAGCTAAGAGGTTTTAGAGCTAGAAATAGCAAG | | anti-purM | |
| Target-purT-F | | ATACTAGTAACATCACGCGAGTTGCTGCGTTTTAGAGCTAGAAATAGCAAG | | anti-purT | |
| Target-puuA-F | | ATACTAGTGTTCTCTACTTCAACGATATGTTTTAGAGCTAGAAATAGCAAG | | anti-puuA | |
| Target-pykA-F | | ATACTAGTTCCGATGGTGCAAACAATTTGTTTTAGAGCTAGAAATAGCAAG | | anti-pykA | |
| Target-pykF-F | | ATACTAGTTTTTGTTCTGCGAAGCCTTCGTTTTAGAGCTAGAAATAGCAAG | | anti-pykF | |
| Target-pyrG-F | | ATACTAGTCAGAGAGGATACGACCCCGCGTTTTAGAGCTAGAAATAGCAAG | | anti-pyrG | |
| Target-pyrH-F | | ATACTAGTAGCAGAATGCGTTTATAGACGTTTTAGAGCTAGAAATAGCAAG | | anti-pyrH | |
| Target-queC-F | | ATACTAGTTGCCTGCACCAGACAGGTGGGTTTTAGAGCTAGAAATAGCAAG | | anti-queC | |
| Target-ravA-F | | ATACTAGTTTCTTTCCGCTAATAAATGAGTTTTAGAGCTAGAAATAGCAAG | | anti-ravA | |
| Target-rbsB-F | | ATACTAGTTAGCGCAACAGCGGAAACCAGTTTTAGAGCTAGAAATAGCAAG | | anti-rbsB | |
| Target-rbsC-F | | ATACTAGTACGGCGACCAGAGACAGTCTGTTTTAGAGCTAGAAATAGCAAG | | anti-rbsC | |
| Target-rbsA-F | | ATACTAGTATTTAACGCTGCGCCCGAGAGTTTTAGAGCTAGAAATAGCAAG | | anti-rbsA | |
| Target-rbsK-F | | ATACTAGTTAATGCTGCCAAGAACAACGGTTTTAGAGCTAGAAATAGCAAG | | anti-rbsK | |
| Target-recQ-F | | ATACTAGTTTGTAAAACCTGTTTAGCTCGTTTTAGAGCTAGAAATAGCAAG | | anti-recQ | |
| Target-relA-F | | ATACTAGTGCACTCACACGACTTCTGGCGTTTTAGAGCTAGAAATAGCAAG | | anti-relA | |
| Target-rep-F | | ATACTAGTCGACAGCTTGTTGTTGGCCGGTTTTAGAGCTAGAAATAGCAAG | | anti-rep | |
| Target-rfaE-F | | ATACTAGTACGCCTGCACGTTCAAACGCGTTTTAGAGCTAGAAATAGCAAG | | anti-rfaE | |
| Target-rhaB-F | | ATACTAGTGACGGCGACACAATTGCGAAGTTTTAGAGCTAGAAATAGCAAG | | anti-rhaB | |
| Target-rhlB-F | | ATACTAGTTTCTACAACCTTCGGATGCAGTTTTAGAGCTAGAAATAGCAAG | | anti-rhlB | |
| Target-ribF-F | | ATACTAGTCACACCCTTCTTGCGGGGCCGTTTTAGAGCTAGAAATAGCAAG | | anti-ribF | |
| Target-rimK-F | | ATACTAGTCGTTCCATCCCGGGACAATAGTTTTAGAGCTAGAAATAGCAAG | | anti-rimK | |
| Target-rtcA-F | | ATACTAGTGCTGGCCGGTTATCATCGACGTTTTAGAGCTAGAAATAGCAAG | | anti-rtcA | |
| Target-sapD-F | | ATACTAGTAGGTTACGAATATCGAGTAAGTTTTAGAGCTAGAAATAGCAAG | | anti-sapD | |
| Target-sapB-F | | ATACTAGTCAATAACAAAATGCGGCGTAGTTTTAGAGCTAGAAATAGCAAG | | anti-sapB | |
| Target-sapC-F | | ATACTAGTTCGCTGTATACGCTATCGTAGTTTTAGAGCTAGAAATAGCAAG | | anti-sapC | |
| Target-sapF-F | | ATACTAGTGAAACCAGCCGGTCCGGTAGGTTTTAGAGCTAGAAATAGCAAG | | anti-sapF | |
| Target-sbcD-F | | ATACTAGTGATGCCAGTCTGAGGTGTGAGTTTTAGAGCTAGAAATAGCAAG | | anti-sbcD | |
| Target-sbcC-F | | ATACTAGTGTTGCTGGCGAACGGCTCGCGTTTTAGAGCTAGAAATAGCAAG | | anti-sbcC | |
| Target-selD-F | | ATACTAGTACCAGCTCCGTGGCTGTATTGTTTTAGAGCTAGAAATAGCAAG | | anti-selD | |
| Target-serS-F | | ATACTAGTGGCTCATTACGCAGCAGATTGTTTTAGAGCTAGAAATAGCAAG | | anti-serS | |
| Target-spoT-F | | ATACTAGTAGGTTTGAATCAGTTGATTCGTTTTAGAGCTAGAAATAGCAAG | | anti-spoT | |
| Target-srkA-F | | ATACTAGTTGGTATCCGGGTGTAGTGTCGTTTTAGAGCTAGAAATAGCAAG | | anti-srkA | |
| Target-ssuB-F | | ATACTAGTTGAGCAACAATGGCGTGCCCGTTTTAGAGCTAGAAATAGCAAG | | anti-ssuB | |
| Target-ssuC-F | | ATACTAGTCGCAATAACCACTTCTTCACGTTTTAGAGCTAGAAATAGCAAG | | anti-ssuC | |
| Target-ssuA-F | | ATACTAGTTATACGTAACGCTTCAGGCGGTTTTAGAGCTAGAAATAGCAAG | | anti-ssuA | |
| Target-sucC-F | | ATACTAGTCGGTGCTGGTAAGCCATAGCGTTTTAGAGCTAGAAATAGCAAG | | anti-sucC | |
| Target-sucD-F | | ATACTAGTAAAGCCCTGGCAGATAACCTGTTTTAGAGCTAGAAATAGCAAG | | anti-sucD | |
| Target-tauB-F | | ATACTAGTTGCCGGTTTGCCGCCATAATGTTTTAGAGCTAGAAATAGCAAG | | anti-tauB | |
| Target-tauC-F | | ATACTAGTAAGGTCACCTGACGCGAGAGGTTTTAGAGCTAGAAATAGCAAG | | anti-tauC | |
| Target-tauA-F | | ATACTAGTAAAAGCGATGAATGCCAGTGGTTTTAGAGCTAGAAATAGCAAG | | anti-tauA | |
| Target-tbpA-F | | ATACTAGTGCCGCGCACAGGAGTAAAAGGTTTTAGAGCTAGAAATAGCAAG | | anti-tbpA | |
| Target-*thiP*-F | | ATACTAGTAGCCGGGAATTAACGGCTGAGTTTTAGAGCTAGAAATAGCAAG | | anti-thiP | |
| Target-thiQ-F | | ATACTAGTCGGCAAATGGTGGTAAAGCCGTTTTAGAGCTAGAAATAGCAAG | | anti-thiQ | |
| Target-tcdA-F | | ATACTAGTCGCCAGCGCTTCCGCCGCCCGTTTTAGAGCTAGAAATAGCAAG | | anti-tcdA | |
| Target-tdcD-F | | ATACTAGTCAGTTAATAACCAAAACAACGTTTTAGAGCTAGAAATAGCAAG | | anti-tdcD | |
| Target-tdk-F | | ATACTAGTAGACTTACCCGCATTCATTGGTTTTAGAGCTAGAAATAGCAAG | | anti-tdk | |
| Target-thiD-F | | ATACTAGTACCACCACTCGGATCAGTACGTTTTAGAGCTAGAAATAGCAAG | | anti-thiD | |
| Target-thiF-F | | ATACTAGTGGCTATAACGCATAAAGTCAGTTTTAGAGCTAGAAATAGCAAG | | anti-thiF | |
| Target-thiI-F | | ATACTAGTTAAAGCGCAAGCGCACAGATGTTTTAGAGCTAGAAATAGCAAG | | anti-thiI | |
| Target-thiK-F | | ATACTAGTATGGGATTATTGCTGCGAAAGTTTTAGAGCTAGAAATAGCAAG | | anti-thiK | |
| Target-thiL-F | | ATACTAGTGTCAAAATAACGGGCAATCAGTTTTAGAGCTAGAAATAGCAAG | | anti-thiL | |
| Target-thiM-F | | ATACTAGTCAGATTGCGCTGAACCCAGCGTTTTAGAGCTAGAAATAGCAAG | | anti-thiM | |
| Target-thrA-F | | ATACTAGTGGCATTGCTTTCCAGAATATGTTTTAGAGCTAGAAATAGCAAG | | anti-thrA | |
| Target-thrB-F | | ATACTAGTCATATTGGCACTGGAAGCCGGTTTTAGAGCTAGAAATAGCAAG | | anti-thrB | |
| Target-thrS-F | | ATACTAGTCCATCAGGAAGAGTTATAACGTTTTAGAGCTAGAAATAGCAAG | | anti-thrS | |
| Target-tilS-F | | ATACTAGTGGCCACCAGAATCTGGCGTGGTTTTAGAGCTAGAAATAGCAAG | | anti-tilS | |
| Target-tmcA-F | | ATACTAGTCCCCGCTCAACACCAGCAAGGTTTTAGAGCTAGAAATAGCAAG | | anti-tmcA | |
| Target-tmk-F | | ATACTAGTCTCAACCACCACATTACGCGGTTTTAGAGCTAGAAATAGCAAG | | anti-tmk | |
| Target-trpS-F | | ATACTAGTTGTGCGCCACTAAAAACGATGTTTTAGAGCTAGAAATAGCAAG | | anti-trpS | |
| Target-tsaC-F | | ATACTAGTGAAAACGGCTTCCGTTGGATGTTTTAGAGCTAGAAATAGCAAG | | anti-tsaC | |
| Target-ttcA-F | | ATACTAGTAATCATATTGAAGTCAGCAAGTTTTAGAGCTAGAAATAGCAAG | | anti-ttcA | |
| Target-tyrS-F | | ATACTAGTCGCTTCCTCGTCCGTCACCTGTTTTAGAGCTAGAAATAGCAAG | | anti-tyrS | |
| Target-udk-F | | ATACTAGTACTGGCAATAAGACTCTTGCGTTTTAGAGCTAGAAATAGCAAG | | anti-udk | |
| Target-ugpB-F | | ATACTAGTGCTGAAGCTGTATAATGTAAGTTTTAGAGCTAGAAATAGCAAG | | anti-ugpB | |
| Target-ugpE-F | | ATACTAGTTGAATATCGTCAGCCACGGAGTTTTAGAGCTAGAAATAGCAAG | | anti-ugpE | |
| Target-ugpA-F | | ATACTAGTAGCGCGAGCGGAACACCGGAGTTTTAGAGCTAGAAATAGCAAG | | anti-ugpA | |
| Target-ugpC-F | | ATACTAGTGGTCAGCGGTTTAATGACCTGTTTTAGAGCTAGAAATAGCAAG | | anti-ugpC | |
| Target-valS-F | | ATACTAGTGCGGCTGTTCGATATCTTGTGTTTTAGAGCTAGAAATAGCAAG | | anti-valS | |
| Target-waaP-F | | ATACTAGTCCACGCCAAAGTGTGGCAAGGTTTTAGAGCTAGAAATAGCAAG | | anti-waaP | |
| Target-waaY-F | | ATACTAGTCGTTCGTTACGCTTAACTTTGTTTTAGAGCTAGAAATAGCAAG | | anti-waaY | |
| Target-wzc-F | | ATACTAGTATCACTGCCCGTTACCGGAGGTTTTAGAGCTAGAAATAGCAAG | | anti-wzc | |
| Target-xylB-F | | ATACTAGTGAGTGGATGCGGGCGCGAAAGTTTTAGAGCTAGAAATAGCAAG | | anti-xylB | |
| Target-xylF-F | | ATACTAGTAAGCAGGAGTGAGGTGCAAAGTTTTAGAGCTAGAAATAGCAAG | | anti-xylF | |
| Target-xylH-F | | ATACTAGTACGGCCAATTTCACTTCAGAGTTTTAGAGCTAGAAATAGCAAG | | anti-xylH | |
| Target-xylG-F | | ATACTAGTCTTCACACTGCCGAAGGTTTGTTTTAGAGCTAGAAATAGCAAG | | anti-xylG | |
| Target-ybcF-F | | ATACTAGTCGCCTCACCGCGCTGGAGTAGTTTTAGAGCTAGAAATAGCAAG | | anti-ybcF | |
| Target-ybhF-F | | ATACTAGTCAGGCCGTTCAGCGTGATAAGTTTTAGAGCTAGAAATAGCAAG | | anti-ybhF | |
| Target-ybhR-F | | ATACTAGTGCAACGACTGCAACTCTTTGGTTTTAGAGCTAGAAATAGCAAG | | anti-ybhR | |
| Target-ybhS-F | | ATACTAGTCGCGACGCCAGGACAGGATCGTTTTAGAGCTAGAAATAGCAAG | | anti-ybhS | |
| Target-ycaO-F | | ATACTAGTCCAGAGCGGCATCTTTGCCGGTTTTAGAGCTAGAAATAGCAAG | | anti-ycaO | |
| Target-ychF-F | | ATACTAGTAGGGTAGATTTCCCGACGTTGTTTTAGAGCTAGAAATAGCAAG | | anti-ychF | |
| Target-yeaG-F | | ATACTAGTCCTTGGCAGCTTCATATCGCGTTTTAGAGCTAGAAATAGCAAG | | anti-yeaG | |
| Target-yegS-F | | ATACTAGTATTAAGAATCAGTAAGCTGGGTTTTAGAGCTAGAAATAGCAAG | | anti-yegS | |
| Target-yehW-F | | ATACTAGTAGAGCAATGAGCCAGAACAGGTTTTAGAGCTAGAAATAGCAAG | | anti-yehW | |
| Target-yehX-F | | ATACTAGTATTGAGATTGAGATCGTTAAGTTTTAGAGCTAGAAATAGCAAG | | anti-yehX | |
| Target-yehY-F | | ATACTAGTGCGCCAGAACAGGATTAATAGTTTTAGAGCTAGAAATAGCAAG | | anti-yehY | |
| Target-osmF-F | | ATACTAGTCCTGCCCAGAGCTTTAAGAGGTTTTAGAGCTAGAAATAGCAAG | | anti-osmF | |
| Target-yejA-F | | ATACTAGTGGGTTCGCCCAGCACGGCAAGTTTTAGAGCTAGAAATAGCAAG | | anti-yejA | |
| Target-yejE-F | | ATACTAGTCCCAACGGGCCTGATTGACGGTTTTAGAGCTAGAAATAGCAAG | | anti-yejE | |
| Target-yejB-F | | ATACTAGTCCAGCAACAGACGGCGAATCGTTTTAGAGCTAGAAATAGCAAG | | anti-yejB | |
| Target-yejF-F | | ATACTAGTCTGTACGTACGGTTTGCTGAGTTTTAGAGCTAGAAATAGCAAG | | anti-yejF | |
| Target-yihV-F | | ATACTAGTGTAATAGATGCGATCCATCAGTTTTAGAGCTAGAAATAGCAAG | | anti-yihV | |
| Target-ytfQ-F | | ATACTAGTGGCCAACGCCATAGACGACAGTTTTAGAGCTAGAAATAGCAAG | | anti-ytfQ | |
| Target-ytfT-F | | ATACTAGTGTGGTGTCCGGAAGAGATTGGTTTTAGAGCTAGAAATAGCAAG | | anti-ytfT | |
| Target-ytfR-F | | ATACTAGTGAGGATCTCCTGGTGTTGGTGTTTTAGAGCTAGAAATAGCAAG | | anti-ytfR | |
| Target-yjfF-F | | ATACTAGTACGCCGATGGTGATCATCAGGTTTTAGAGCTAGAAATAGCAAG | | anti-yjfF | |
| Target-zntA-F | | ATACTAGTGCTTTCTTGCCGTGATTGTCGTTTTAGAGCTAGAAATAGCAAG | | anti-zntA | |
| Target-znuA-F | | ATACTAGTTGCACCTCCCCAGAGAGCGGGTTTTAGAGCTAGAAATAGCAAG | | anti-znuA | |
| Target-znuB-F | | ATACTAGTATGATCCCGGCTAACCAACCGTTTTAGAGCTAGAAATAGCAAG | | anti-znuB | |
| Target-znuC-F | | ATACTAGTCATCAGAGAGGACGCGGCGTGTTTTAGAGCTAGAAATAGCAAG | | anti-znuC | |
| TargetF-R | | CGGAATTCGGAAGATCTAAAAAAAGCACCGACTCG | | PCR for N20-sgRNA | |
| 5F-yahk | GCAGGATGACGCCAGCTTTGTGCTGGTGGAC | | deletion of *yahk* | |  |
| 5R-yahk | TTAATGATATCATTGTGTTTACTCCTGATTAGCTATGTGTATTTGGCAACG | |  | |  |
| 3F- yahk | AGGAGTAAACACAATGATATCATTAAAAGCTCCGCACAATAATTTG | |  | |  |
| 3R- yahk | TATTCAAGAAGAATACATTTGCAATCTGTTCGCACTGAATCGATAAACTG | |  | |  |
| 5F-fecE | TTTTTAGAAAAATCGATGGTCAGAAACTGGATTAGCAATTCCGTTCC | | deletion of *fecE* | |  |
| 5R-fecE | CTTGTGAGAATGCGATAAATTGCACAGGCCGTAAGAACCAAACCACG | |  | |  |
| 3F-fecE | CTTACGGCCTGTGCAATTTATCGCATTCTCACAAGCAACCAGACAAACCACGGC | |  | |  |
| 3R-fecE | CGGATTATCTGATGCTCTCGCGCCCGCAGGATGTGAACAACGCCCTG | |  | |  |
| 5F-pabA | AACTGCACACCTTCCAGATCCCACTGGCGATG | | replacement of the native promoter of *yahk* | |  |
| 5R-pabA | CAAAAGATCTTTTCGGAATTAAGGAGGTAATAAATATGATCCTGCTTATAGATAACTAC | |  | |  |
| QSF-pabA | CTCCTTAATTCCGAAAAGATCTTTTGAATTCGCTAAACAACTGAAGCCATTG | |  | |  |
| QSR-pabA | CAATGGGGGATCTCACCGCAACGCGTCTTACATGAAAAAGGTTCTTG | |  | |  |
| 3F-pabA | CAATGGGGGATCTCACCGCAACGCGTCTTACATGAAAAAGGTTCTTG | |  | |  |
| 3R-pabA | CATGTAAGACGCGTTGCGGTGAGATCCCCCATTGCCCCACTCTGATTTGCCTG | |  | |  |
| qcysG-F | TTGTCGGCGGTGGTGATGTC | | qPCR for *cysG* | |  |
| qcysG-R | ATGCGGTGAACTGTGGAATAAACG | |  | |  |
| qaraH-F | CATCATTTCAGACGGTAAAGCGG | | qPCR for *araH* | |  |
| qaraH-R | CCAAAGATAATCAGACACGCGAC | |  | |  |
| qfecE-F | GAGTTAATGTCGCCATGAACCAG | | qPCR for *fecE* | |  |
| qfecE-R | GCTCATCAAGTAATACAACGGGC | |  | |  |
| qyahK-F | GATCACATGACCGGCACCTATAA | | qPCR for *yahK* | |  |
| qyahK-R | CGAATACGCAGAACATATCGCTC | |  | |  |
| qyqjH-F | CAGCTGTATGTCTGCGATGAATC | | qPCR for *yqjH* | |  |
| qyqjH-R | CGAGATAATCCTGACAGGCGTTA | |  | |  |


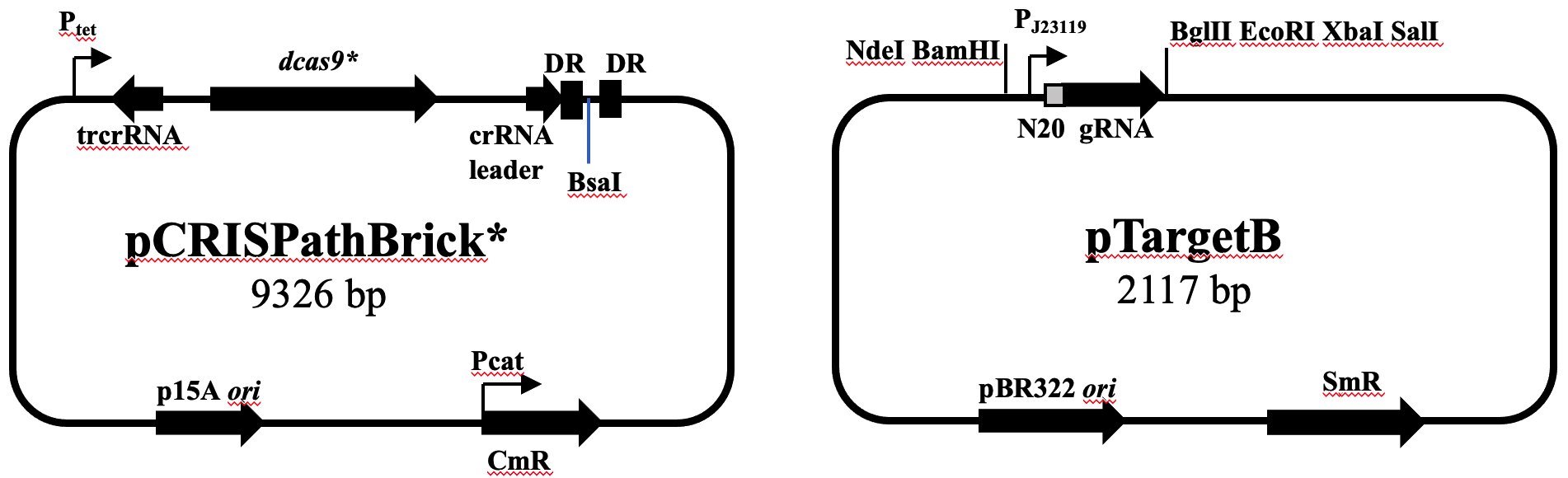


**Supplementary Figure 6 Plasmid maps used for CRISPRi in this study**

**References**

1. Livak KJ, Schmittgen TD. Analysis of relative gene expression data using real-time quantitative PCR and the 2(T)(-Delta Delta C) method. Methods. 2001;25(4):402-8.
